# Comparative analysis of the immune repertoire between peripheral blood and bone marrow fluids in those infected by EBV and immunodeficiency: A retrospective case study

**DOI:** 10.1097/MD.0000000000039501

**Published:** 2024-09-20

**Authors:** Mei Yue, Juanjuan Li, Junhui Li, Tao Hu, Shunqiao Feng, Jing Cao, Ruihong Tang, Pengpeng Wang, Fengjiao Zhu, Lu Han, Jian Wu, Xiaodai Cui, Rong Liu

**Affiliations:** a Department of Hematology, Children’s Hospital of Capital Institute of Pediatrics, Beijing, China; b MyGenostics Inc, Beijing, China; c Department of Key Laboratory, Children’s Hospital of Capital Institute of Pediatrics, Beijing, China.

**Keywords:** B cell receptor, Epstein-Barr virus, immune repertoire, immunodeficiency, T cell receptor

## Abstract

High-throughput immune repertoire (IR) sequencing provides direct insight into the diversity of B cell receptor (BCR) and T cell receptor (TCR), with great potential to revolutionize the diagnosis, monitoring, and prevention of immune system–related disorders. In this study, multiplex PCR was applied to amplify the complementarity-determining regions of BCR and TCR, followed by comprehensive analysis by high-throughput sequencing. We compare the TCR (BCR) of bone marrow fluid (BMF) and peripheral blood (PB) samples from 17 patients in the Epstein-Barr and immunodeficiency groups, respectively. Our study shows that the diversity of the IR of blood samples is very similar to that of bone marrow samples statistically. However, the distributions of the monoclonal genes are significantly different in these 2 samples of most patients. This suggests that the BMFs can be replaced by the PB samples in diversity detection of IR to monitor the immune status of the body, while the detection of the BMFs is unreplaceable when the monoclonal change occurs. We used high-throughput sequencing to assess the TCR and BCR of the patients and provide a basis for the clinical analysis of PB and bone marrow samples and selection of disease diagnosis and monitoring methods.

## 1. Introduction

Immune repertoire (IR) refers to the combined collection of T cell receptors (TCRs) and B cell receptors (BCRs, also known as immunoglobulins) present in the circulatory system of an individual at a given time.^[1]^ TCRs and BCRs are composed of multiple peptide chains with antigen specificity, and the complementarity-determining regions (CDRs), including CDR1, CDR2, and CDR3, exhibit significant diversity in amino acid composition and sequence order, resulting in a vast library of TCRs and BCRs that enable the selective adaptive immune response.^[2]^ In recent IR research, there has been increasing focus on the study of CDR3 diversity,^[3,4]^ which is shaped during lymphocyte maturation through rearrangement of V, D, and J genes, as well as DNA base single nucleotide polymorphisms and insertion/deletion mutations.^[5–7]^ High-throughput sequencing (HTS) techniques have revolutionized the field of IR research, allowing for efficient and large-scale determination of complementary regions of lymphocyte receptors.^[8]^

Deep profiling of CDR3s using HTS has emerged as a powerful approach for elucidating the composition and distribution of CDR3s in a given sample, providing detailed sequence-level information.^[4]^ Epstein-Barr virus (EBV) is the first human cancer virus discovered and is associated with the development of various hematopoietic system tumors, epithelial cell tumors, mesenchymal tumors, and particularly hematologic tumors/lymphomas.^[9,10]^ EBV is highly prevalent in the human population, with over 90% of adults being infected. The clinical outcome of EBV infection depends on the balance between viral and immune functions, and it can lead to chronic active infection,^[11]^ nasopharyngeal carcinoma,^[12]^ and hematological malignancies.^[13]^ The host mounts an antiviral immune response, involving both innate and adaptive effector functions, in response to primary EBV infection.^[14]^ The EBV-specific TCR repertoire has been reported in lung transplant recipients,^[15]^ primary infection,^[16]^ and common variable immune deficiency, but there are limited reports on the analysis of IR in children with EBV infection.^[17–19]^ Given the advantages of IR in evaluating immune function and detecting clonal changes, it is necessary to study and compare the IR of bone marrow fluid (BMF) and peripheral blood (PB) samples in patients with EBV infection and analyze the potential interchangeability between these 2 sample types. In this study, T lymphocytes and B lymphocytes were selected as the research targets, and multiplex PCR technology was used to amplify the CDR regions of TCRs and BCRs, followed by HTS technology for a comprehensive assessment of the diversity of the immune system. The TCR and BCR IR of BMF and PB samples were compared to investigate their similarities and differences.

## 2. Methods

### 2.1. Patients and ethics statement

The study recruited a total of 17 children, with 12 children in the Epstein-Barr (EB) group (P01–P12) and 5 children with immunodeficiency (P13–P17) in the immunodeficiency group from unrelated Chinese families. Of the total participants, 5 were female and 12 were male, with a mean age of 4.25 years (ranging from 3 months to 10.75 years). The patients were selected from the Children’s Hospital of the Capital Institute of Pediatrics from 2018 to 2021.

In the EB group, there were 7 cases of chronic active EBV infection in patients P01, P02, P03, P04, P07, P08, and P12. There was 1 case of c-Myc-positive precursor B lymphocytic leukemia with EBV infection complicated by hemophagocytic syndrome in patient P10. In addition, there were 3 cases of EBV-associated hemophagocytic syndrome in patients P05, P06, and P09 and 1 case of X-linked immunodeficiency with magnesium deficiency EBV infection and tumorigenesis, along with EBV-positive mucosa–associated lymphoid tissue extranodal marginal zone B cell lymphoma in patient P11.

In the immunodeficiency group, there were 3 cases of severe combined immune deficiency in patients P13, P14, and P15, 1 case of hyper IgM syndrome in patient P16, and 1 case of T lymphoblastic lymphoma in patient P17. Detailed clinical data for each patient can be found in the Supplement Clinical Data, Supplemental Digital Content, http://links.lww.com/MD/N595, and Table S1, Supplemental Digital Content, http://links.lww.com/MD/N597.

The study collected 2 mL of PB and 5 mL of BMF from both the EB group and immunodeficiency group for TCR and BCR IR analysis using next-generation sequencing. The sample collection time and method for each patient can be found in the Supplementary Material.

Furthermore, whole exon sequencing was performed in all patients and their parents, and Sanger sequencing verification was conducted. Details of sample collection, exon sequencing, and Sanger sequencing methods can be found in the Supplementary Methods, Supplemental Digital Content, http://links.lww.com/MD/N596. This study was conducted in accordance with the Declaration of Helsinki. It is important to note that this study has been approved by the Ethics Committee of the Children’s Hospital of Capital Institute of Pediatrics, with the reference number SHERLLM2022023. Prior to sample collection, written informed consent was obtained from the parents or legal guardians of the patients. All patients and their families also received genetic counseling, and all data analyzed in this study were anonymized to protect patient privacy.

### 2.2. Sequencing analysis of immune group library

VDJ genes of 17 patients were captured and sequenced with high-throughput sequencing. The bioinformatics analysis of the patient’s immune group database includes Shannon entropy, V/J usage, and monoclonal status. The overall process can be divided into the following steps: collect PB and BMF and send samples to Mygenostics (Beijing, China); DNA extraction, using the extracted DNA as a template; multiplex PCR amplifies the VDJ region to obtain the antigen receptor gene region; purify the amplified PCR products and build a library; and the library is put on the computer for HTS. Illumina NextSeq500 sequencer 2*150; bioinformatics analysis to evaluate the diversity of the IR. The detailed information analysis steps are given as follows: use Cutadapt to remove low quality and sequence with connectors, basic statistics, and quality control (QC). The QC standard of nucleic acid extraction and library establishment is >20 ng/UL according to the glue diagram to determine the risk experiment. Data QC will fine-filter raw reads to produce clean reads. The data volume of normal samples reached 3G, Q30 reached more than 90%, and the number of reads was >3 million. The clean sequence is compared with the reference sequence. The reference sequence comes from all the V/D/J sequences in the ImMunoGeneTics database; count the frequency information of V/D/J genes according to the comparison results.

### 2.3. Data comparison analysis

The purpose of the data comparison step is to identify the specific VDJ combinations from the sequenced reads and determine the CDR3 region, as well as the relative abundance of each clone combination. The ImMunoGeneTics database (http://www.imgt.org/) is a comprehensive resource that contains nucleic acid sequence data related to the immune system, including 66 V genes, 2 D genes, and 13 J genes. The CDR3 region is known to be the most variable region of the BCR/TCR protein, as it is responsible for antigen and antibody binding. It typically consists of 10 to 20 amino acids and is located between the conserved cysteine (Cys, C) at the end of the V region and phenylalanine (Phe, F) or tryptophan (Trp, W) in the J region.

### 2.4. Clonal diversity analysis

Cloning refers to the same type of BCR/TCR multiplied by the same type of B/T cells. We define a clone with the same V/J gene and the same amino acid sequence in the CDR3 region. Diversity is a measure of the variation of organisms. For the immune system, the higher the diversity of immune cells, the more stable the system, and the stronger the resistance to antigens; on the contrary, if there are only a few clones in the immune system, the diversity of the immune system is very low, indicating that there may be diseases, infections, and immunity. The reaction occurs. Assessing and measuring the diversity of the immune system provide a quantitative indicator of health.

We regard each different clone as a different species, and the number of sequences in each clone as the abundance of the species is measured by the Shannon entropy and the inverse Simpson index. The method of Shannon entropy and inverse Simpson index calculated can be found in the Supplementary Methods, Supplemental Digital Content, http://links.lww.com/MD/N596.

### 2.5. V/J Usage

Each immune protein in the IR has very little structural difference from each other, but there are many types of subtypes. It is this diversity that plays a vital role in the maintenance of health; the more the subtypes of immune proteins (VDJ combination), the more effective it is to resist pathogens, and the fewer the subtypes (the fewer types of VDJ combinations), the easier it is to contract diseases.

### 2.6. Statistical analysis

We used GraphPad Prism software (version 6.0) and SPSS (version 20.0) to analyze our data. *P* < .05 were considered statistically significant.

## 3. Results

### 3.1. Patient and Shannon entropy results

The results of routine blood tests revealed that the total number of lymphocytes in the blood of most patients was lower than the normal range. Further analysis through exome sequencing identified pathogenic mutations in 3 of 17 patients. Patient P13 had a spliced mutation (c.115 + 2T > C) in the IL-2RG gene, P14 had a missense mutation (c.202G > A) in the IL-2RG gene, and P15 had 2 inframe mutations (c.449dupT and c.192-195delinsA) in the DNA crosslinking repair 1C (DCLRE1C) gene, as shown in Table S1, Supplemental Digital Content, http://links.lww.com/MD/N597.

In addition, BCR and TCR IR sequencing results showed that the Shannon entropy values for BCR and TCR were not within the normal range in 53% of the patients, as indicated in Table S2, Supplemental Digital Content, http://links.lww.com/MD/N597. Specifically, Shannon entropy results indicated that the diversity of clones was poor in these patients, suggesting potential abnormalities in the IR.

### 3.2. V/J gene usage

**Figure 1.**
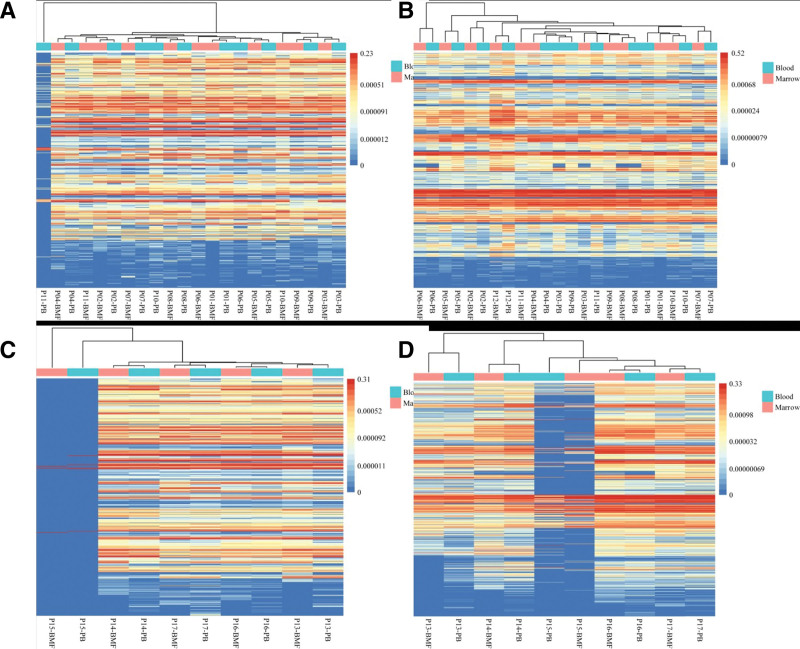
Comparison of V/J gene usage between peripheral blood and bone marrow fluid. (A) Representative treemaps of the V/J gene usage of B cell receptor (BCR) in the Epstein-Barr (EB) group. (B) Representative treemaps of the V/J gene usage of T cell receptor (TCR) in the EB group. (C) Representative treemaps of the V/J gene usage of BCR in the immunodeficiency group. (D) Representative treemaps of the V/J gene usage of TCR in the immunodeficiency group. The X-axis is the patient number. The Y-axis is the proportion of cloned sequences.

Based on the heat map clustering analysis, it was observed that the EB group and immunodeficiency group did not show a clear distinction between PB and BMF samples based on VJ gene frequency. The samples from the bone marrow and blood of the same patient were found to be adjacent to each other in the representative treemaps. This suggests that there may not be significant differences in VJ gene usage between PB and BMF samples in these groups, as revealed by the heat map clustering analysis. Further analysis and interpretation of the results can provide insights into the IR characteristics of the EB group and immunodeficiency group in relation to VJ gene usage in different sample types (Fig. 1).

### 3.3. Similarity assessment of BCR and TCR-VJ gene combination of bone marrow and PB samples

**Figure 2.**
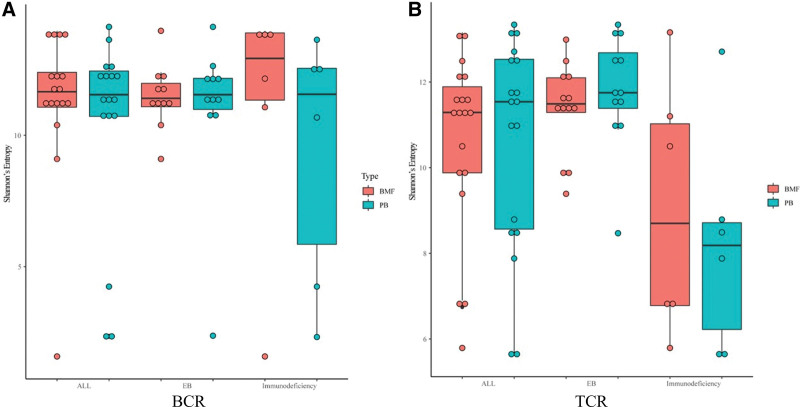
Box plots of clonal diversity of peripheral blood and bone marrow samples. (A) Box plots of clonal diversity of peripheral blood and bone marrow samples in B cell receptor (BCR). (B) Box plots of clonal diversity of peripheral blood and bone marrow samples in T cell receptor (TCR). The X-axis is the group name. The Y-axis is the Shannon entropy. BMF = bone marrow fluid.

A stacked bar chart of VJ gene usage distribution was generated for all patients, and the results showed that the clone diversity of PB and bone marrow samples was highly similar, as depicted in Figures S1 to S17, Supplemental Digital Content, http://links.lww.com/MD/N598. However, in the case of patient 11, who was diagnosed with X-linked immunodeficiency with magnesium deficiency, EBV infection, and tumorigenesis, as well as EBV-positive mucosa–associated lymphoid tissue extranodal marginal zone B cell lymphoma, there were differences observed in BCR-VJ gene usage between the BMF (P11-BMF) and PB (P11-PB) samples. Similarly, in the immunodeficiency group, the BMF and PB samples of most patients showed similar BCR-VJ gene usage, except for patient 15 who was diagnosed with DCLRE1C complex heterozygous mutation by whole exome sequencing, and exhibited differences in BCR-VJ gene usage between the BMF (P15-BMF) and PB (P15-PB) samples. On the other hand, the BMF and PB samples of each person in both the EB group and immunodeficiency group showed similar TCR-VJ gene usage. The differences in the diversity of clones between PB and bone marrow samples were further analyzed using the rank sum test. The results of the rank sum test indicated that there was no significant difference observed. These findings provide insights into the similarity of VJ gene usage distribution between PB and bone marrow samples in the studied groups, with some exceptions in specific patient cases (Fig. 2; Table 1).

**Table 1:** Differences in the diversity of immune repertoire between peripheral blood and bone marrow samples.

Groups	BCR Shannon entropy	TCR Shannon entropy
All	0.724	0.5861
EB group	0.7969	0.3777
Immunodeficiency group	0.3095	0.6905

BCR = B cell receptor, EB = Epstein-Barr, TCR = T cell receptor.

### 3.4. Distribution difference of monoclonal in the bone marrow and PB samples

The distribution of monoclonal antibodies in bone and PB samples of each patient was analyzed using a *t* test. A significance level of *P* > .05 was considered statistically significant. The results revealed that there were significant differences in the monoclonal antibody distribution between PB and bone marrow samples in the majority of patients. Specifically, in most patients, both BCR and TCR monoclonal distributions differed significantly between BMF and PB samples. However, in a total of 6 patients (4 patients in BCR and 2 patients in TCR), no significant differences were observed in the monoclonal antibody distribution between BMF and PB samples (Table 2). These findings highlight the variation in monoclonal antibody distribution between bone marrow and PB samples in different patients, with some cases showing significant differences and others showing similarities.

**Table 2:** Monoclonal differences in peripheral blood and bone marrow samples from each patient.

Patient number	*P* value (*t* test) for TCR	*P* value (*t* test) for BCR
P01	3.65 × 10^−22^	3.65 × 10^−22^
P02	4.49 × 10^−11^	5.69 × 10^−124^
P03	2.61 × 10^−56^	0
P04	6.63 × 10^−9^	2.20 × 10^−16^
P05	2.00 × 10^−107^	2.20 × 10^−16^
P06	.0413	1.26 × 10^−88^
P07	6.63 × 10^−61^	.039388116
P08	3.68 × 10^−14^	.268538647
P09	.004519	.295361
P10	6.68 × 10^−46^	.0000175
P11	6.09 × 10^−9^	0
P12	2.20 × 10^−16^	NA
P13	3.82 × 10^−1^	0
P14	1.12 × 10^−1^	.403716
P15	3.97 × 10^−6^	0
P16	5.87 × 10^−2^	7.69 × 10^−61^
P17	2.66 × 10^−12^	4.01 × 10^−9^

Note: *P* > .05, no differences.

BCR = B cell receptor, NA = unchecked, TCR = T cell receptor.

### 3.5. Consistent immune monitoring with the same kind of sample types

Finally, we assessed the feasibility of immune status monitoring by comparing the BCR and TCR IR in 2 cases (P13 and P14) who underwent bone marrow transplantation. PB samples were selected as the immune monitoring samples after transplantation. In the case of patient 13, the diversity of clones in the immune monitoring samples was significantly lower compared to the PB samples collected before transplantation, specifically in BCR (Figure S13, Supplemental Digital Content, http://links.lww.com/MD/N598). However, the diversity of clones in TCR was similar (Figure S13, Supplemental Digital Content, http://links.lww.com/MD/N598). On the other hand, for patient 14, the diversity of clones in the immune monitoring samples was similar to both the PB and bone marrow samples collected before transplantation, both in BCR and TCR (Figure S14, Supplemental Digital Content, http://links.lww.com/MD/N598). These findings suggest that monitoring PB samples may offer a noninvasive approach for evaluating the diversity of the immune system in patients undergoing bone marrow transplantation.

## 4. Discussion

The diversity of T and B cells, in terms of their receptor sequences, is vast in the vertebrate immune system, providing broad protection against a wide range of pathogens.^[1]^ In this study, we compared the TCR and BCR repertoires of BMF and PB samples in the EB group and the immunodeficiency group. The complexity and heterogeneity of BCR/TCR rearrangement repertoire in lymphocyte-derived cell lines were revealed in 2018, including EBV-infected cells,^[20]^ which changed our understanding of this type of disease, and further observation may require more data. EBV is a common virus in humans, with an infection rate of up to 90% in adults. Asymptomatic infections or infectious mononucleosis are more common in people with normal immune function. Acute infectious mononucleosis, an EBV-induced disease prevalent in young adults but not children, is associated with increased frequencies of T cells cross-reactive to EBV.^[21]^ However, individuals with immune deficiency, such as X-linked lymphoproliferative hyperplasia, early stage after bone marrow transplantation, and postorgan transplantation patients, may present with diseases such as hemophagocytic syndrome and posttransplant lymphoproliferative disease with high mortality and high risk. Chronic active EBV infection is a rare form of primary EBV infection, where, for unknown reasons, EBV continues to be active in the body with flu-like symptoms, accumulates lymphocytes other than B cells, and even produces clonal changes in natural killer (NK) cells and T cells.

In immunosuppressed lung transplant recipients, the EBV-specific CD8 T-Cell receptor αβ repertoire was found to be maintained, and early detection of EBV-specific T cells could potentially serve as a predictor of subsequent EBV blood viremia.^[15]^ In patients with EBV-infected HIV, loss of T cell functionality and TCR-Vβ repertoire against EBV has been associated with poor prognosis and clinical parameters.^[18]^ In our study, we also observed a child with X-linked immunodeficiency associated with enzyme deficiency who had EBV infection and tumor formation in the EB group. At that time, the child had also been diagnosed with EBV-positive mucosa–associated lymphoid tissue extranodal marginal zone B cell lymphoma, and clonal changes were detected when PB was collected for BCR examination. It is worth noting that the child was tested after the second CHOP chemotherapy, which could be related to the incomplete remission of the primary disease after chemotherapy.

We identified a child in our study who had a severe infection after birth and was found to have a heterozygous mutation in the DCLRE1C complex by exome sequencing. DCLRE1C encodes the Artemis protein, which has endonuclease and exonuclease activities and plays a role in the nonhomologous DNA end joining–mediated DNA double-strand break repair pathway. Nonhomologous DNA end joining is the main DNA repair mechanism involved in V(D)J recombination, which is essential for the development of functional T and B cells.^[22]^ Deleterious mutations in DCLRE1C can result in significant impairment of V(D)J recombination, leading to functional defects in T and B cells. The child’s PB T and B cell counts were severely reduced, while NK cell counts were normal, consistent with the manifestations of severe combined immune deficiency with T-B-NK + phenotype. The number of BCR bands in both PB and bone marrow samples was found to be rare, which is likely related to the severe reduction in B cell counts.^[23]^ Our study also compared the TCR and BCR repertoire diversity in PB and bone marrow samples of patients and found that the Shannon quotient, a measure of diversity, showed no significant differences between the 2 sample types for all patients. This suggests that PB samples can be used as a substitute for bone marrow samples in evaluating the diversity of IRs and assessing the immune status of the body.

We also compared the TCR and BCR repertoire similarity between bone marrow and PB samples in 2 bone marrow transplantation patients. Our results showed that the diversity of clones, as indicated by Shannon entropy and V/J gene usage, was similar in most patients. However, for monoclonal genes, the distribution in PB and bone marrow samples differed significantly in most patients, suggesting that PB samples may not be suitable for monitoring changes in monoclonal gene populations in the body. These findings are particularly useful for observing changes in the condition of children who are infected with EBV and may provide insights into the prognosis of the disease. Further research is needed to better understand the dynamics of TCR and BCR repertoire diversity in different sample types and clinical conditions and optimize the use of PB samples for IR analysis in various contexts.

The evaluation of immune status through TCR and BCR repertoire analysis in patients undergoing bone marrow transplantation is an important focus of our study. In 2 cases (P13 and P14), we observed that the diversity of clones in immune monitoring samples from patient 13 was significantly lower in the bone marrow sample compared to the PB sample collected before transplantation, which may be attributed to a decrease in corresponding B and T cells in the bone marrow. However, in all other cases, the clone diversity of PB and bone marrow samples was highly similar. This suggests that monitoring IR diversity and status can be effectively conducted using only PB samples, which is clinically more convenient and noninvasive.

## 5. Conclusion

Our findings indicate that PB samples may play a more practical and consistent role in immune monitoring compared to bone marrow samples. This has important implications for clinical practice, as PB sampling is less invasive and more feasible for longitudinal monitoring of patients’ immune status. However, there are some limitations to our study. First, the sample size is relatively small, and further validation with a larger sample size is needed to confirm our conclusions. In addition, our results were based on next-generation sequencing, and results from other detection methods should be considered to further evaluate our findings. Nevertheless, our study provides a basis for the clinical analysis of PB and bone marrow samples in evaluating immune status and monitoring IR diversity in patients undergoing bone marrow transplantation. Further research is warranted to expand and validate these findings in larger cohorts and with different methodologies.

## Acknowledgments

The authors wish to thank all the patients who participated in the study and all the staff at the clinic for their help in recruiting patients.

## Author contributions

**Validation:** Fengjiao Zhu, Pengpeng Wang.

**Data curation:** Mei Yue.

**Formal analysis:** Mei Yue.

**Project administration:** Mei Yue.

**Writing – original draft:** Mei Yue.

**Investigation:** Juanjuan Li, Junhui Li, Tao Hu, Shunqiao Feng, Jing Cao, Ruihong Tang.

**Resources:** Juanjuan Li, Junhui Li, Tao Hu, Shunqiao Feng, Jing Cao, Ruihong Tang.

**Software:** Lu Han.

**Visualization:** Jian Wu.

**Funding acquisition:** Xiaodai Cui, Rong Liu.

**Writing – review & editing:** Xiaodai Cui, Rong Liu.

## Supplementary Material


