# Supplementary material for: Comparative analysis of the immune repertoire between peripheral blood and bone marrow fluids in those infected by EBV and immunodeficiency: A retrospective case study
Source: Medicine (Baltimore). 2024 Sep 20;103(38):e39501. doi: 10.1097/MD.0000000000039501 (PMC11419465; doi:10.1097/MD.0000000000039501)
Supplement: Supplementary file 1 [file medi-103-e39501-s001.docx]

**Supplementary clinical data**

**Comparative analysis of the immune repertoire between peripheral blood and bone marrow fluids in EBV infected children and children with immunodeficiency: A retrospective case study**

**This PDF file includes:**

- Supplementary clinical data for 17 patients described in this manuscript (P01-P17, Page1-Page3)

###### Supplementary clinical data for 17 patients described in this manuscript

1. **P01** The patient, a male, aged 10.66 years, was diagnosed with chronic active EBV infection. The duration of the disease was 21 months. At the beginning of the disease, the HLH-94 regimen was used for chemotherapy, intermittent fever and rash were observed, and the whole exon sequencing showed no abnormalities. After six months of drug withdrawal, the fever recurred. The copy number of EBV was 10e4/ mL, ferritin was normal, ALT 106.8U/L, AST148.9/L, and LR2R 1024U/ mL.
2. **P02** The patient, a male, aged 1 year, was diagnosed with chronic active EBV infection. The duration of the disease was 2 months with intermittent fever and lymphadenopathy. The copy number of EBV was 10e3/ mL by nucleic acid test, and no immune deficiency gene was found by whole exon sequencing.
3. **P03** The patient, a male, aged 7 years, was diagnosed with chronic active EBV infection. The duration of the disease was 6 months with intermittent fever and rash. The copy number of EBV was 10e3/ mL by nucleic acid test, and no immune deficiency gene was found by whole exon sequencing.
4. **P04** This patient, a female, aged 5 years, with intermittent fever for 20 days was diagnosed with EBV-associated hemophagocytic syndrome. In the fifth week of treatment with the HLH-94 regimen, the EBV copy number was 10e7/mL, and no immune deficiency was found by whole exon sequencing.
5. **P05** This patient, female, aged 5 years, with intermittent fever for 20 days was diagnosed with EBV associated hemophagocytic syndrome. In the fifth week of treatment with HLH-94 regimen, EBV copy number was 10e7/mL, and no immune deficiency were found by whole exon sequencing.
6. **P06** This patient, a female, aged 7 years, with intermittent fever for 17 days was diagnosed with EBV-associated hemophagocytic syndrome. In the second week of treatment with the HLH-94 regimen, the EBV copy number was 10e5/mL, and no immune deficiency was found by whole exon sequencing.
7. **P07** This patient, a female, aged 14 months, with intermittent fever for more than 10 days and lymphadenectasis swelled for more than 4 months, was diagnosed with chronic active EBV infection. EBV copy number was 10e4/mL, and no immune deficiency was found by whole exon sequencing.
8. **P08** This patient, male, aged 7 years, with intermittent fever and rash for 2 months, was diagnosed with chronic active EBV infection. EBV copy number was 10e5/mL, and no immune deficiency was found by whole exon sequencing.
9. **P09** This patient, a male, aged 4 years, with intermittent fever for 1 week, was diagnosed with EBV-associated hemophagocytic syndrome. EBV copy number was 10e6/mL, and no immune deficiency was found by whole exon sequencing.
10. **P10** This patient, male, aged 4 years, with rash for 5 days and abnormal blood routine for 5 days, was diagnosed with CMYC positive precursor B lymphocytic leukemia (leukemia) with EBV infection complicated and hemophagocytic syndrome. EBV copy number was 10e6/mL. At the beginning of the disease, the Inter-B-NHL2010 regimen was used for chemotherapy.
11. **P11** This patient, male, aged 9 years, EBV positive MALT lymphoma was diagnosed more than 3 months. After 3 rounds of R-CHOP, PET/CT showed complete remission. EBV copy number was 10e3/mL. Whole exon sequencing revealed X-linked immunodeficiency with magnesium deficiency EBV infection and tumourigenesis and EBV-positive mucosa-associated lymphoid tissue extranodal marginal zone B cell lymphoma.

XMEN is a combined immunodeficiency disorder, but this patient had reduced numbers of CD8+T and NK cells. The patient's course of illness is relatively mild compared to SCID and is generally not associated with severe life-threatening infections. Decreased expression of the receptor NKG2D on NK cells and CD8+T cells is typical in these patients. NKG2D is an important activation receptor of CD8+T cells against EBV cytotoxicity, and the loss of NKG2D expression seriously affects the clearance of EB virus in vivo. XMEN patients are more likely to present with lymphoproliferative disease or EB virus-associated lymphoma after EB virus infection. Therefore, we think XMEN is more suitable in EB group[1].

1. **P12** This patient, a female, aged 3.75 years, fever and neck mass of more than 3 months, was diagnosed with chronic active EBV infection. EBV copy number was 10e3/mL. Patients were treated with HLH-94 and DEP regimen successively and no immune deficiency was found by whole exon sequencing.
2. **P13** This patient, male, aged 5 months, was diagnosed with severe combined immune deficiency (SCID). Whole exon sequencing revealed splice site mutation of IL-2RG gene (de novo mutation). The classic manifestation of this mutation is the loss of T cells and NK cells, a normal number of B cells but lack of function. Before transplantation, the peripheral blood lymphocyte count of the patient was 67*10e9/L, flow cytometry indicated abnormal T lymphocytes, TCR rearrangement was positive, cellular immune T lymphocytes increased and 97% were CD8+T, absolute value of NK cells decreased significantly, and B lymphocytes decreased slightly. Humoral immunity showed IgA below the lower limit and normal IgG and IgM levels. On January 14, 2020, umbilical cord blood transfusion was performed during immune reconstructive surgery (bone marrow and peripheral blood samples were collected before surgery). On February 28, 2020, peripheral blood samples were collected and immune repertoire tested on the 45th day after surgery. At the same day, the total number of lymphocytes was 1.68*10e9/L, the number of T lymphocytes was 1400/ul which lower than normal, the number of B lymphocytes was 0, and the NK cell count was normal.
3. **P14** This patient, male, aged 2.22 years, was diagnosed with severe combined immune deficiency (SCID). The patient had tuberculous brain abscess. Whole exon sequencing revealed splice site mutation of IL-2RG gene (de novo mutation). Before transplantation, the peripheral blood lymphocyte count of the patient was 2.91*10e9/L, the cell immune T lymphocyte count was 1581/ul, CD4+T count was 382/uL, absolute value of NK cells decreased significantly, and B lymphocytes count was normal. Humoral immunity showed IgA below the lower limit and normal IgG and IgM levels. On November 1, 2019, umbilical cord blood transfusion was performed during immune reconstructive surgery (bone marrow and peripheral blood samples were collected before surgery). On April 10, 2020, peripheral blood samples were collected and immune repertoire tested on the 163th day after surgery. At the same day, the total number of lymphocytes was 1.38*10e9/L, the number of T lymphocytes was 1102/uL which lower than normal, CD4+T count was 259/uL, the number of B lymphocytes was 170/uL, and the NK cell count was101/uL.
4. **P15** This patient, male, aged 3 months, was diagnosed with severe combined immune deficiency (SCID). The peripheral blood lymphocyte count of the patient was 0.17*10e9/L, the T and B lymphocyte counts were severely reduced, and the NK cell count was 181/uL. On August 3, 2020, umbilical cord blood transfusion was performed during immune reconstructive surgery (bone marrow and peripheral blood samples were collected before surgery). On September 25, 2020, 39 days after the surgery, the peripheral blood lymphocyte count of the patient was 0.68*10e9/L, the T lymphocyte counts was 444/uL, the B lymphocyte counts was 6/uL, were severely reduced, and the NK cell count was 184/uL.
5. **P16** This patient, male, aged 4 years, recurrent oral ulcers after birth, was diagnosed with hyper IgM syndrome. Humoral immunoassay showed IgG value was 0.152g/L (normal range was 5.53-13.07 g/L), IgA value <0.0647g/L (normal range was 0.33-1.8g/L), IgM value was 2.89g/L (normal range was 5.06-2.18g/L). Whole exon sequencing showed frameshift mutation (c.158_161dupTAGA pE54fs*4) which mutation from his mother. Blood and bone marrow samples were taken for immune repertoire testing before the immune reconstructive surgery and the absolute value of lymphocyte was 4.02*10e9/L in blood routine examination on the same day.
6. **P17** This patient, female, aged 10.75 years, was diagnosed with T lymphoblastic lymphoma stage III. The patient was treated with COG AALL 1231 T-LLY regimen, and imaging showed complete remission of the lesion. Developed severe myelosuppression after chemotherapy and extreme granulocytopenia in the peripheral blood, which lasted for 4 weeks, with pulmonary aspergillus infection. In this case, we performed an immune repertoire examination to further assess the patient's immune function status. The patient was assigned to the immunodeficiency group mainly because he was in a severe secondary immunodeficiency state at the time.
