# Supplementary material for: Comparative analysis of the immune repertoire between peripheral blood and bone marrow fluids in those infected by EBV and immunodeficiency: A retrospective case study
Source: Medicine (Baltimore). 2024 Sep 20;103(38):e39501. doi: 10.1097/MD.0000000000039501 (PMC11419465; doi:10.1097/MD.0000000000039501)
Supplement: Supplementary file 2 [file medi-103-e39501-s002.docx]

**Table S1 Patient clinical data and whole exon test results**

| Group | Patient No. | Diagnose | Gender | Age | PB Total lymphocyte count | | WES Result |
| --- | --- | --- | --- | --- | --- | --- | --- |
|  |  |  |  |  | B cells count（/ul） | T cells count（/ul） |  |
| EB | P01 | EBV infection(CAEBV) | male | 10y8m | 0 | 1750 | NA |
| EB | P02 | EBV infection(CAEBV) | male | 1y | 1090.82 | 3594.77 | NA |
| EB | P03 | EBV infection(CAEBV) | male | 7y | 327 | 1545.87 | NA |
| EB | P04 | EBV infection(CAEBV) | male | 3y | 2.53*10E9/L | | NA |
| EB | P05 | EBV associated  hemophagocytic syndrome | female | 5y | 118.55 | 1503.09 | NA |
| EB | P06 | EBV associated hemophagocytic syndrome | female | 7y | 129.78 | 1020.02 | NA |
| EB | P07 | EBV infection(CAEBV) | female | 1y2m | 166.76 | 4805.21 | NA |
| EB | P08 | EBV infection(CAEBV) | male | 7y | 477.32 | 968.25 | NA |
| EB | P09 | EBV associated hemophagocytic syndrome | male | 4y | 128.36 | 480.54 | NA |
| EB | P10 | CMYC positive precursor B lymphocytic leukemia (leukemia) with EBV infection complicated and hemophagocytic syndrome | male | 4y | 0.33*10E9/L | | NA |
| EB | P11 | X linked immunodeficiency with magnesium deficiency EBV infection and tumourigenesis and EBV positive mucosa associated lymphoid tissue extranodal marginal zone B cell lymphoma | male | 9y | 0.65*10E9/L | | NA |
| EB | P12 | EBV infection(CAEBV) | female | 3y9m | 713.9 | 1848 | NA |
| Immunodeficiency | P13 | SCID （severe combined immune deficiency） | male | 5m | 406 | 39499 | c.115+2T>C |
| Immunodeficiency | P14 | SCID （severe combined immune deficiency） | male | 2y3m | 3.32*10E9/L | | c.202G>A; p.E68K |
| Immunodeficiency | P15 | SCID （severe combined immune deficiency） | male | 3m | 0.46 | 1.53 | c.449dupT; c.192-195delinsA |
| Immunodeficiency | P16 | hyper IgM syndrome | male | 4y | 4.02*10E9/L | | NA |
| Immunodeficiency | P17 | T lymphoblastic lymphoma | female | 10y9m | 0.61*10E9/L | | NA |

Note: NA: No pathogenic variation was detected; Total lymphocyte count, B cells count, T cells count from peripheral blood; No cell count was performed on bone marrow.

**Table S2 The Shannon's Entropy of peripheral blood and bone marrow fluids in BCR and TCR**

| Patient No. | BMF sample No. | TCR Shannon's Entropy | BCR Shannon's Entropy | PB sample No. | TCR Shannon's Entropy | BCR Shannon's Entropy | PB sample No. (immune monitoring) | TCR Shannon's Entropy | BCR Shannon's Entropy |
| --- | --- | --- | --- | --- | --- | --- | --- | --- | --- |
| P01 | P01-BMF | 12.49 | ***12.4*** | P01-PB | ***11.49*** | ***14.13*** | - | - | - |
| P02 | P02-BMF | ***9.89*** | 11.03 | P02-PB | ***10.89*** | ***12.64*** | - | - | - |
| P03 | P03-BMF | 12.15 | ***13.98*** | P03-PB | 13.34 | ***12.1*** | - | - | - |
| P04 | P04-BMF | 11.68 | 11.41 | P04-PB | 11.59 | 11.23 | - | - | - |
| P05 | P05-BMF | ***11.49*** | 11.86 | P05-PB | 12.56 | ***12.24*** | - | - | - |
| P06 | P06-BMF | ***9.39*** | ***9.1*** | P06-PB | ***8.47*** | 10.81 | - | - | - |
| P07 | P07-BMF | ***9.87*** | 11.15 | P07-PB | 11.75 | 10.72 | - | - | - |
| P08 | P08-BMF | ***11.29*** | ***12.1*** | P08-PB | 11.75 | 12.06 | - | - | - |
| P09 | P09-BMF | 12.99 | 11.66 | P09-PB | 13.22 | 11.55 | - | - | - |
| P10 | P10-BMF | 12.1 | 10.39 | P10-PB | ***11.07*** | 11.17 | - | - | - |
| P11 | P11-BMF | ***11.36*** | 11.4 | P11-PB | 13.06 | ***2.37*** | - | - | - |
| P12 | P12-BMF | 11.57 | NA | P12-PB | 12.45 | NA | - | - | - |
| P13 | P13-BMF | ***5.79*** | ***13.97*** | P13-PB | ***5.67*** | 10.68 | 19C144830 | 7.88 | ***4.24*** |
| P14 | P14-BMF | ***6.74*** | ***13.7*** | P14-PB | ***8.49*** | ***12.45*** | 19C178169 | 8.79 | ***12.59*** |
| P15 | P15-BMF | ***6.9*** | ***1.58*** | P15-PB | ***5.62*** | ***2.32*** | - | - | - |
| P16 | P16-BMF | 13.16 | ***13.97*** | P16-PB | 12.71 | ***13.64*** | - | - | - |
| P17 | P17-BMF | ***11.2*** | ***12.16*** | P17-PB | ***10.5*** | 11.07 | - | - | - |

Note: -, unchecked; The Shannon entropy range of normal human’s BCR is 10.33~12.09 and TCR is 11.55~14.42. Lower or higher than this range indicates poor diversity of the patient's immune repertoire.
