# Supplementary material for: Comparative analysis of the immune repertoire between peripheral blood and bone marrow fluids in those infected by EBV and immunodeficiency: A retrospective case study
Source: Medicine (Baltimore). 2024 Sep 20;103(38):e39501. doi: 10.1097/MD.0000000000039501 (PMC11419465; doi:10.1097/MD.0000000000039501)
Supplement: Supplementary file 3 [file medi-103-e39501-s003.docx]

**Supplementary Methods**

**Sample collection**

The patient who was diagnosed with EBV infected-related or immunodeficiency related disease by clinician and routine laboratory examination. Peripheral blood and bone marrow samples were collected simultaneously for T and B cell receptor immune repertoire analysis using next-generation sequencing. For patients treated with cord blood transplants, peripheral blood and bone marrow samples are collected before the transplant. When the patient is in a complete chimeric state after transplant, collecting the “immune monitoring” samples (peripheral blood sample).

Bone marrow fluids sample collection method: We use a bone marrow puncture to extract the bone marrow, collected 2 ml bone marrow fluid from a normal aspirate by EDTA Routine blood tube (Anticoagulant tube), and transported to genetic testing center within 2 hours at room temperature.

Peripheral blood sample collection method: We collected 5ml of peripheral blood from the patient's upper arm by EDTA Routine blood tube (Anticoagulant tube), and transported to genetic testing center within 2 hours at room temperature.

**Whole Exon Sequencing and Sanger Sequencing**

Genomic DNA was extracted using a QIAamp Blood Midi Kit (QIAGEN, Valencia, CA). An Illumina NextSeq 500 sequencer (Illumina, San Diego, CA, USA) was used with 150 bp paired-end reads following the manufactures instructions. After sequencing, the raw data were saved in FASTQ format. Quality control (QC) filters were applied to remove reads with low quality. Then, the clean reads were assembled and spliced using the second-generation sequencing analysis platform provided by MyGenostics and the coverage and sequencing quality of the target region were evaluated. Finally, flash analysis platform was used to analyze the pathogenicity of variation, and the possible variation loci were determined. The pathogenicity of variation loci was also analyzed according to ACMG (American College of Medical Genetics and Genomics) genetic variation classification criteria and guidelines. An ABI3730xl sequencer (Applied Biosystems, USA) was used to apply the Sanger sequencing method, and the results were compared to the capture sequencing results to confirm the candidate mutation and assess their inheritance patterns through familial segregation studies.

**Shannon Entropy and Inverse Simpson’s Index calculated**

ImmuneSeq software was used for determination of productive clonality, clonal sharing, CDR3β, and CDR3H length, and percent productiveness. Clonality is calculated as 1-normalized Shannon's entropy. This measures how evenly receptor sequences are distributed amongst a set of T or B cells, the Shannon entropy range of normal human’s BCR is 10.33~12.09 and TCR is 11.55~14.42. The greater the number of diversity of immune cells and the higher Shannon entropy in the normal range, the stronger the immune response ability of human body to external invasion, the immune system is about stable. Lower or higher than this range indicates poor diversity of the patient's immune repertoire. Shannon's H, which measures the overall diversity in a given population, and takes into account the number of unique sequences (richness of the repertoire) and how evenly the sequences are distributed, was calculated using the following formulas:

$$Shannon^{'}s Entropy= -\sum_{i}^{n} p_{i} \times\log_{2} p_{i}$$

i = Clone i

n= The total number of cloning

$p_{i}$= Proportion of the total sequences belonging to the ‘i’ th unique rearrangement

The Simpson diversity index describes the probability that clones of the same species will be obtained from two consecutive samples of the measured clonal population. It reflects the status and role of amplified clones in the clone population. If the proportion of amplified clones in the clone population is large, the proportion of other non-clonal clones will be reduced, and the Simpson index will be large, indicating that the clone diversity is low. The higher the Simpson index, the lower the diversity of clones. The inverse Simpson diversity index is the inverse of Simpson index, and the greater the inverse Simpson index, the higher the diversity of clones, was calculated using the following formulas:

$$\frac{1}{\lambda}=\frac{1}{\sum_{i=1}^{R} P^{i}}=2_{D}$$

λ= Simpson diversity index

R= Total number of clone species

i = Clone i

$p_{i}$= Proportion of the total sequences belonging to the ‘i’ th unique rearrangement
