# Supplementary material for: Comparative analysis of the immune repertoire between peripheral blood and bone marrow fluids in those infected by EBV and immunodeficiency: A retrospective case study
Source: Medicine (Baltimore). 2024 Sep 20;103(38):e39501. doi: 10.1097/MD.0000000000039501 (PMC11419465; doi:10.1097/MD.0000000000039501)
Supplement: Supplementary file 4 [file medi-103-e39501-s004.pdf]

Supplementary table and figure file legends

Supplementary Clinical data for 17 patients described in this manuscript.

Figure S1. Similarity in clone diversity of peripheral blood and bone marrow fluids samples from patient 01. Stacked Bar Chart of VJ gene: similarity assessment of VJ gene combination of bone marrow and blood samples in BCR (A) and in TCR (D). The abscissa is the sample type; the ordinate is VJ gene sequence proportion. T cell receptor, TCR; B cell receptor, BCR. Bone marrow fluids, BMF; Peripheral blood, PB. (B) VJ gene usage distribution map of the peripheral blood in BCR. (C) VJ gene usage distribution map of the bone marrow fluids in BCR. (E) VJ gene usage distribution map of the peripheral blood in TCR. (F) VJ gene usage distribution map of the bone marrow fluids in TCR. The X-axis is the V gene, Y-axis is the J gene, Z-axis is the number of read.

Figure S2. Similarity in clone diversity of peripheral blood and bone marrow fluids samples from patient 02. Stacked Bar Chart of VJ gene: similarity assessment of VJ gene combination of bone marrow and blood samples in BCR (A) and in TCR (D). The abscissa is the sample type; the ordinate is VJ gene sequence proportion. T cell receptor, TCR; B cell receptor, BCR. Bone marrow fluids, BMF; Peripheral blood, PB. (B) VJ gene usage distribution map of the peripheral blood in BCR. (C) VJ gene usage distribution map of the bone marrow fluids in BCR. (E) VJ gene usage distribution map of the peripheral blood in TCR. (F) VJ gene usage distribution map of the bone marrow fluids in TCR. The X-axis is the V gene, Y-axis is the J gene, Z-axis is the number of read.

Figure S3. Similarity in clone diversity of peripheral blood and bone marrow fluids samples from patient 03. Stacked Bar Chart of VJ gene: similarity assessment of VJ gene combination of bone marrow and blood samples in BCR (A) and in TCR (D). The abscissa is the sample type; the ordinate is VJ gene sequence proportion. T cell receptor, TCR; B cell receptor, BCR. Bone marrow fluids, BMF; Peripheral blood, PB. (B) VJ gene usage distribution map of the peripheral blood in BCR. (C) VJ gene usage distribution map of the bone marrow fluids in BCR. (E) VJ gene usage distribution map of the peripheral blood in TCR. (F) VJ gene usage distribution map of the bone marrow fluids in TCR. The X-axis is the V gene, Y-axis is the J gene, Z-axis is the number of read.

Figure S4. Similarity in clone diversity of peripheral blood and bone marrow fluids samples from patient 04. Stacked Bar Chart of VJ gene: similarity assessment of VJ gene combination of bone marrow and blood samples in BCR (A) and in TCR (D). The abscissa is the sample type; the ordinate is VJ gene sequence proportion. T cell receptor, TCR; B cell receptor, BCR. Bone marrow fluids, BMF; Peripheral blood, PB. (B) VJ gene usage distribution map of the peripheral blood in BCR. (C) VJ gene usage distribution map of the bone marrow fluids in BCR. (E) VJ gene usage distribution map of the peripheral blood in TCR. (F) VJ gene usage distribution map of the bone marrow fluids in TCR. The X-axis is the V gene, Y-axis is the J gene, Z-axis is the number of read.

Figure S5. Similarity in clone diversity of peripheral blood and bone marrow fluids samples from P05. Stacked Bar Chart of VJ gene: similarity assessment of VJ gene combination of bone marrow and blood samples in BCR (A) and in TCR (D). The abscissa is the sample type; the ordinate is VJ gene sequence proportion. T cell receptor, TCR; B cell receptor, BCR. Bone marrow fluids, BMF; Peripheral blood, PB. (B) VJ gene usage distribution map of the peripheral blood in BCR. (C) VJ gene usage distribution map of the bone marrow fluids in BCR. (E) VJ gene usage distribution map of the peripheral blood in TCR. (F) VJ gene usage distribution map of the bone marrow fluids in TCR. The X-axis is the V gene, Y-axis is the J gene, Z-axis is the number of read.

Figure S6. Similarity in clone diversity of peripheral blood and bone marrow fluids samples from patient 06. Stacked Bar Chart of VJ gene: similarity assessment of VJ gene combination of bone marrow and blood samples in BCR (A) and in TCR (D). The abscissa is the sample type; the ordinate is VJ gene sequence proportion. T cell receptor, TCR; B cell receptor, BCR. Bone marrow fluids, BMF; Peripheral blood, PB. (B) VJ gene usage distribution map of the peripheral blood in BCR. (C) VJ gene usage distribution map of the bone marrow fluids in BCR. (E) VJ gene usage distribution map of the peripheral blood in TCR. (F) VJ gene usage distribution map of the bone marrow fluids in TCR. The X-axis is the V gene, Y-axis is the J gene, Z-axis is the number of read.

Figure S7. Similarity in clone diversity of peripheral blood and bone marrow fluids samples from patient 07. Stacked Bar Chart of VJ gene: similarity assessment of VJ gene combination of bone marrow and blood samples in BCR (A) and in TCR (D). The abscissa is the sample type; the ordinate is VJ gene sequence proportion. T cell receptor, TCR; B cell receptor, BCR. Bone marrow fluids, BMF; Peripheral blood, PB. (B) VJ gene usage

distribution map of the peripheral blood in BCR. (C) VJ gene usage distribution map of the bone marrow fluids in BCR. (E) VJ gene usage distribution map of the peripheral blood in TCR. (F) VJ gene usage distribution map of the bone marrow fluids in TCR. The X-axis is the V gene, Y-axis is the J gene, Z-axis is the number of read.

Figure S8. Similarity in clone diversity of peripheral blood and bone marrow fluids samples from patient 08. Stacked Bar Chart of VJ gene: similarity assessment of VJ gene combination of bone marrow and blood samples in BCR (A) and in TCR (D). The abscissa is the sample type; the ordinate is VJ gene sequence proportion. T cell receptor, TCR; B cell receptor, BCR. Bone marrow fluids, BMF; Peripheral blood, PB. (B) VJ gene usage distribution map of the peripheral blood in BCR. (C) VJ gene usage distribution map of the bone marrow fluids in BCR. (E) VJ gene usage distribution map of the peripheral blood in TCR. (F) VJ gene usage distribution map of the bone marrow fluids in TCR. The X-axis is the V gene, Y-axis is the J gene, Z-axis is the number of read.

Figure S9. Similarity in clone diversity of peripheral blood and bone marrow fluids samples from patient 09. Stacked Bar Chart of VJ gene: similarity assessment of VJ gene combination of bone marrow and blood samples in BCR (A) and in TCR (D). The abscissa is the sample type; the ordinate is VJ gene sequence proportion. T cell receptor, TCR; B cell receptor, BCR. Bone marrow fluids, BMF; Peripheral blood, PB. (B) VJ gene usage distribution map of the peripheral blood in BCR. (C) VJ gene usage distribution map of the bone marrow fluids in BCR. (E) VJ gene usage distribution map of the peripheral blood in TCR. (F) VJ gene usage distribution map of the bone marrow fluids in TCR. The X-axis is the V gene, Y-axis is the J gene, Z-axis is the number of read.

Figure S10. Similarity in clone diversity of peripheral blood and bone marrow fluids samples from patient 10. Stacked Bar Chart of VJ gene: similarity assessment of VJ gene combination of bone marrow and blood samples in BCR (A) and in TCR (D). The abscissa is the sample type; the ordinate is VJ gene sequence proportion. T cell receptor, TCR; B cell receptor, BCR. Bone marrow fluids, BMF; Peripheral blood, PB. (B) VJ gene usage distribution map of the peripheral blood in BCR. (C) VJ gene usage distribution map of the bone marrow fluids in BCR. (E) VJ gene usage distribution map of the peripheral blood in TCR. (F) VJ gene usage distribution map of the bone marrow fluids in TCR. The X-axis is the V gene, Y-axis is the J gene, Z-axis is the number of read.

Figure S11. Similarity in clone diversity of peripheral blood and bone marrow fluids samples from patient 11. Stacked Bar Chart of VJ gene: similarity assessment of VJ gene combination of bone marrow and blood samples in BCR (A) and in TCR (D). The abscissa is the sample type; the ordinate is VJ gene sequence proportion. T cell receptor, TCR; B cell receptor, BCR. Bone marrow fluids, BMF; Peripheral blood, PB. (B) VJ gene usage distribution map of the peripheral blood in BCR. (C) VJ gene usage distribution map of the bone marrow fluids in BCR. (E) VJ gene usage distribution map of the peripheral blood in TCR. (F) VJ gene usage distribution map of the bone marrow fluids in TCR. The X-axis is the V gene, Y-axis is the J gene, Z-axis is the number of read.

Figure S12. Similarity in clone diversity of peripheral blood and bone marrow fluids samples from patient 12. Stacked Bar Chart of VJ gene: similarity assessment of VJ gene combination of bone marrow and blood samples in BCR (A). The abscissa is the sample type; the ordinate is VJ gene sequence proportion. T cell receptor, TCR; B cell receptor, BCR. Bone marrow fluids, BMF; Peripheral blood, PB. (B) VJ gene usage distribution map of the peripheral blood in BCR. (C) VJ gene usage distribution map of the bone marrow fluids in BCR.

Figure S13. Similarity in clone diversity of peripheral blood and bone marrow fluids samples from patient 13. Stacked Bar Chart of VJ gene: similarity assessment of VJ gene combination of bone marrow and blood samples in BCR (A) and in TCR (E). The abscissa is the sample type; the ordinate is VJ gene sequence proportion. T cell receptor, TCR; B cell receptor, BCR. Bone marrow fluids, BMF; Peripheral blood, PB. (B) VJ gene usage distribution map of the peripheral blood in BCR. (C) VJ gene usage distribution map of the bone marrow fluids in BCR. (D) VJ gene usage distribution map of the peripheral blood of patients after immune reconstitution in BCR. (F) VJ gene usage distribution map of the peripheral blood in TCR. (G) VJ gene usage distribution map of the bone marrow fluids in TCR. (H) VJ gene usage distribution map of the peripheral blood of patients after immune reconstitution in TCR. The X-axis is the V gene, Y-axis is the J gene, Z-axis is the number of read.

Figure S14. Similarity in clone diversity of peripheral blood and bone marrow fluids samples from patient 14. Stacked Bar Chart of VJ gene: similarity assessment of VJ gene combination of bone marrow and blood samples in BCR (A) and in TCR (E). The abscissa is the sample type; the ordinate is VJ gene sequence proportion. T cell receptor, TCR; B cell

receptor, BCR. Bone marrow fluids, BMF; Peripheral blood, PB. (B) VJ gene usage distribution map of the peripheral blood in BCR. (C) VJ gene usage distribution map of the bone marrow fluids in BCR. (D) VJ gene usage distribution map of the peripheral blood of patients after immune reconstitution in BCR. (F) VJ gene usage distribution map of the peripheral blood in TCR. (G) VJ gene usage distribution map of the bone marrow fluids in TCR. (H) VJ gene usage distribution map of the peripheral blood of patients after immune reconstitution in TCR. The X-axis is the V gene, Y-axis is the J gene, Z-axis is the number of read.

Figure S15. Similarity in clone diversity of peripheral blood and bone marrow fluids samples from patient 15. Stacked Bar Chart of VJ gene: similarity assessment of VJ gene combination of bone marrow and blood samples in BCR (A) and in TCR (D). The abscissa is the sample type; the ordinate is VJ gene sequence proportion. T cell receptor, TCR; B cell receptor, BCR. Bone marrow fluids, BMF; Peripheral blood, PB. (B) VJ gene usage distribution map of the peripheral blood in BCR. (C) VJ gene usage distribution map of the bone marrow fluids in BCR. (E) VJ gene usage distribution map of the peripheral blood in TCR. (F) VJ gene usage distribution map of the bone marrow fluids in TCR. The X-axis is the V gene, Y-axis is the J gene, Z-axis is the number of read.

Figure S16. Similarity in clone diversity of peripheral blood and bone marrow fluids samples from patient 16. Stacked Bar Chart of VJ gene: similarity assessment of VJ gene combination of bone marrow and blood samples in BCR (A) and in TCR (D). The abscissa is the sample type; the ordinate is VJ gene sequence proportion. T cell receptor, TCR; B cell receptor, BCR. Bone marrow fluids, BMF; Peripheral blood, PB. (B) VJ gene usage distribution map of the peripheral blood in BCR. (C) VJ gene usage distribution map of the bone marrow fluids in BCR. (E) VJ gene usage distribution map of the peripheral blood in TCR. (F) VJ gene usage distribution map of the bone marrow fluids in TCR. The X-axis is the V gene, Y-axis is the J gene, Z-axis is the number of read.

Figure S17. Similarity in clone diversity of peripheral blood and bone marrow fluids samples from patient 17. Stacked Bar Chart of VJ gene: similarity assessment of VJ gene combination of bone marrow and blood samples in BCR (A) and in TCR (D). The abscissa is the sample type; the ordinate is VJ gene sequence proportion. T cell receptor, TCR; B cell receptor, BCR. Bone marrow fluids, BMF; Peripheral blood, PB. (B) VJ gene usage distribution map of the peripheral blood in BCR. (C) VJ gene usage distribution map of the

bone marrow fluids in BCR. (E) VJ gene usage distribution map of the peripheral blood in TCR. (F) VJ gene usage distribution map of the bone marrow fluids in TCR. The X-axis is the V gene, Y-axis is the J gene, Z-axis is the number of read.

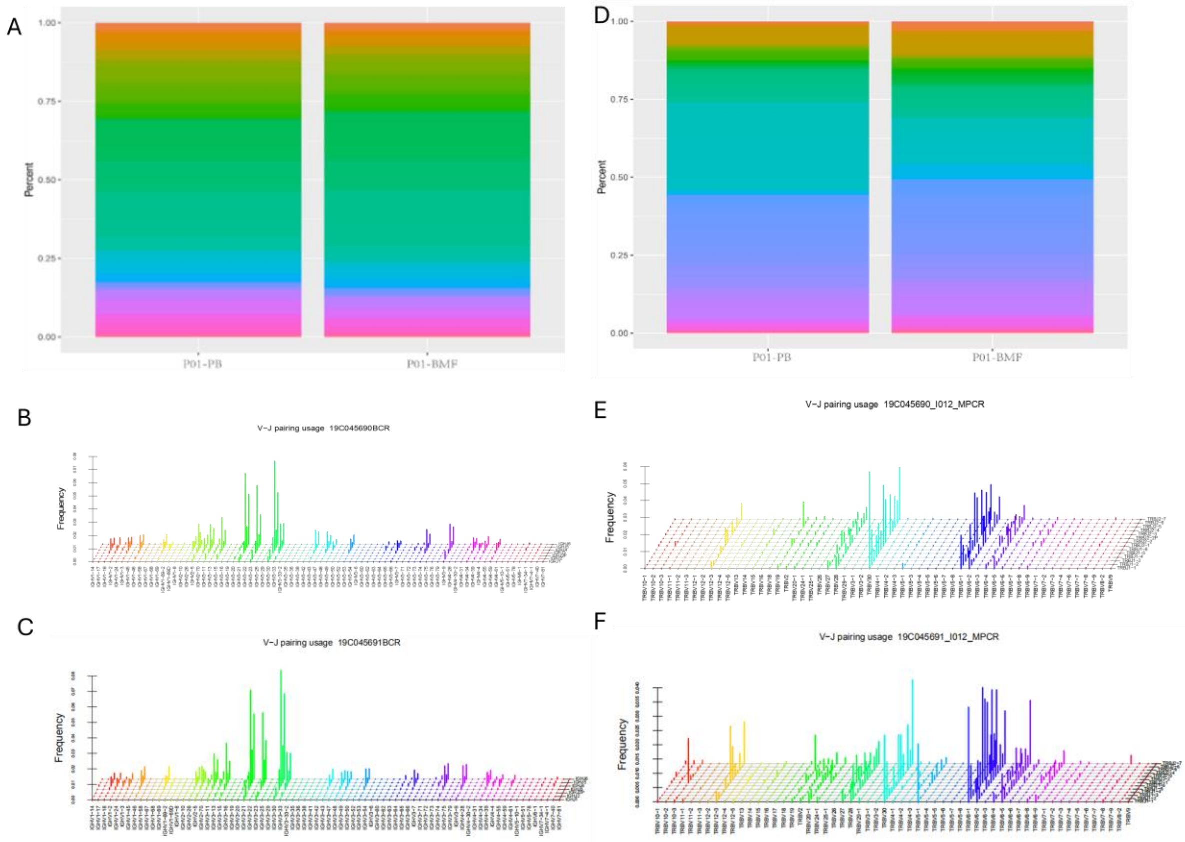
A
D
Percent
1.00
0.75
0.50
0.25
0.00
P01-PB
P01-BMF
B
V−J pairing usage 19C045690BCR
Frequency
C
V−J pairing usage 19C045691BCR
E
V−J pairing usage 19C045690_I012_MPCR
F
V−J pairing usage 19C045691_I012_MPCR

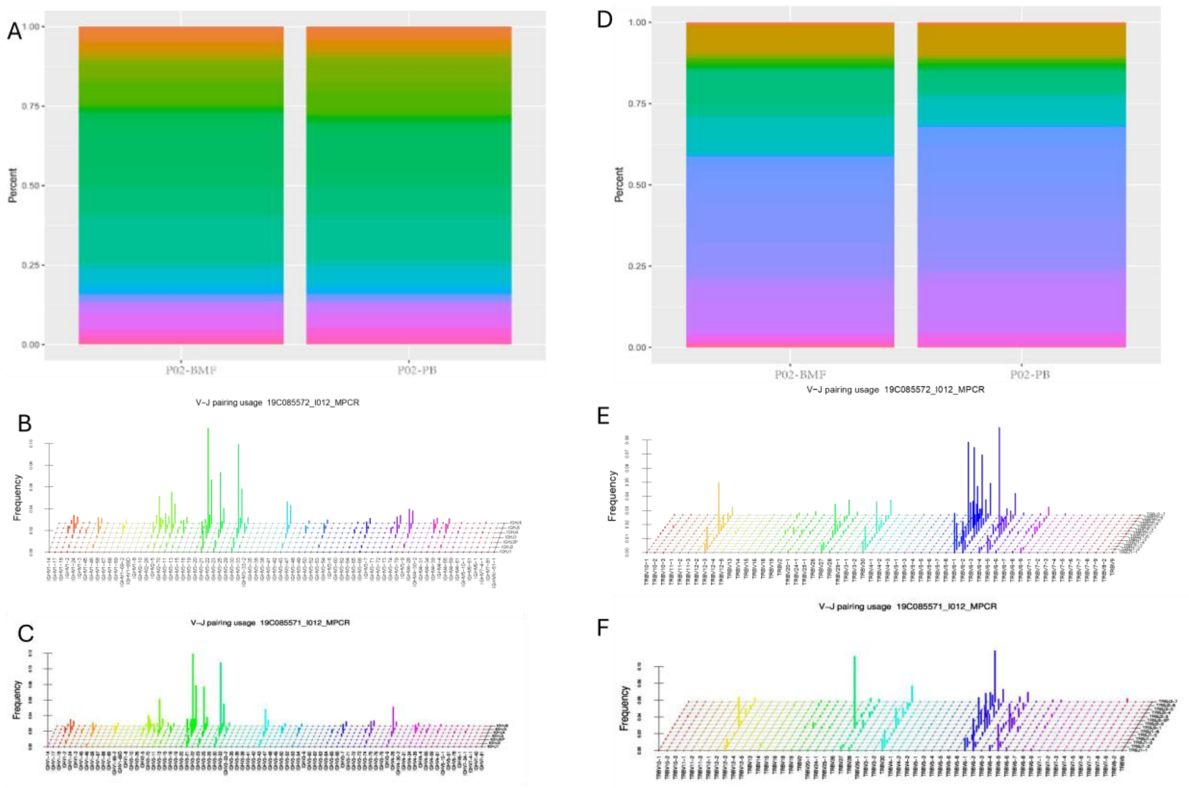

A
1.00
0.75
0.50
0.25
0.00
Percent
P02-BMF
P02-PB
D
Percent
P02-BMF
P02-PB
V-J pairing usage 19C085572_I012_MPCR
B
Frequency
E
Frequency
V-J pairing usage 19C085571_I012_MPCR
C
Frequency
F
Frequency

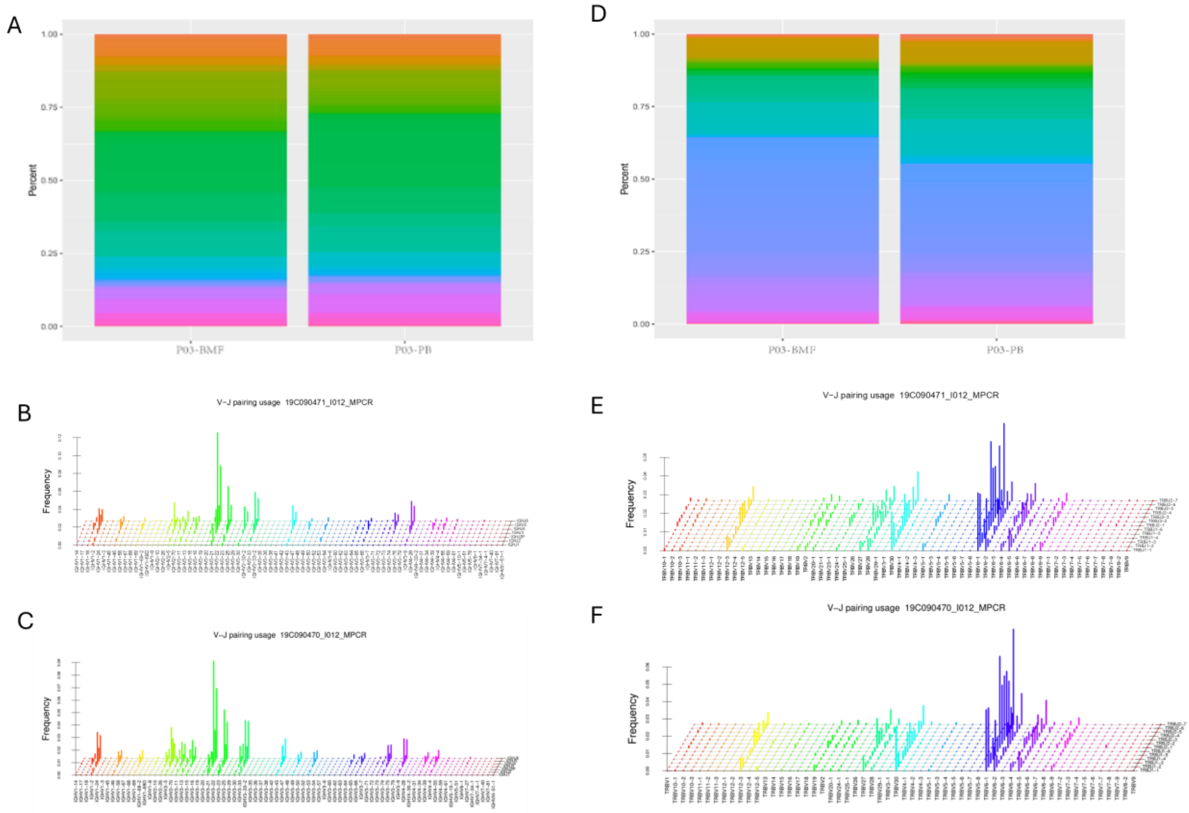
A
1.00
0.75
0.50
0.25
0.00
Percent
P03-BMF
P03-PB
D
1.00
0.75
0.50
0.25
0.00
Percent
P03-BMF
P03-PB
B
V~J pairing usage 19C090471_I012_MPCR
Frequency
E
V~J pairing usage 19C090471_I012_MPCR
Frequency
C
V~J pairing usage 19C090470_I012_MPCR
Frequency
F
V~J pairing usage 19C090470_I012_MPCR
Frequency

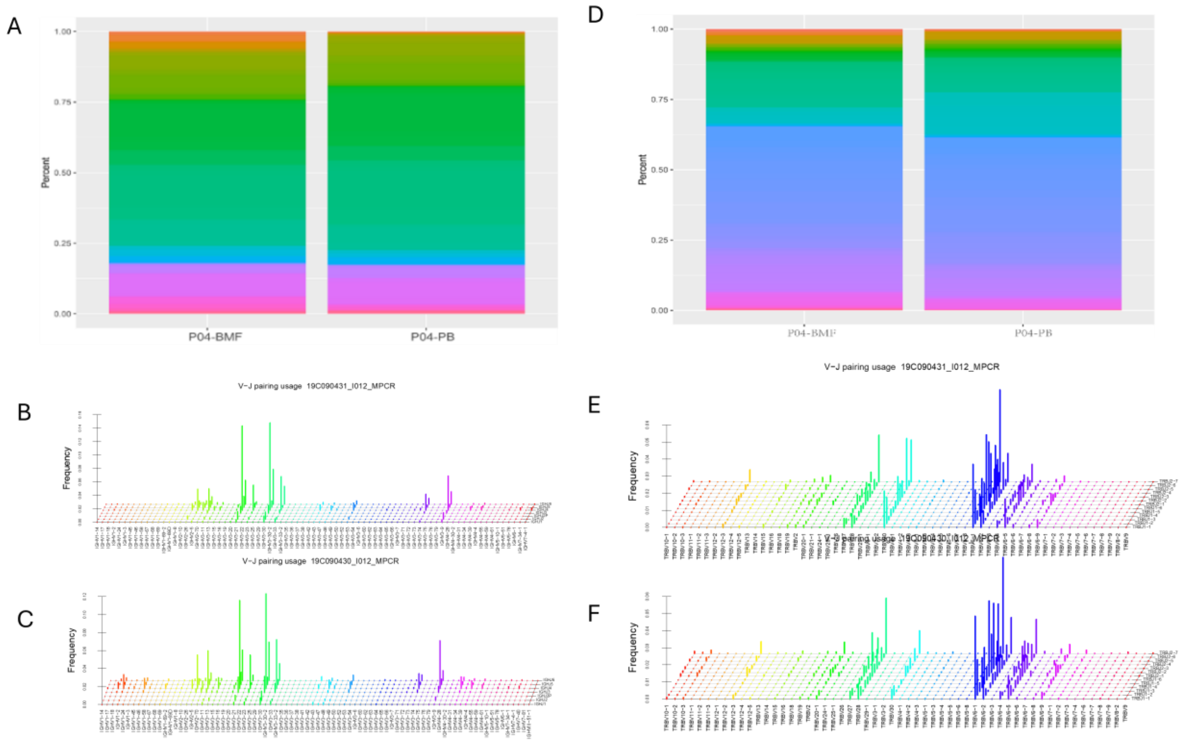
A
B
C
D
E
F
Percent
1.00
0.75
0.50
0.25
0.00
P04-BMF
P04-PB
V-J pairing usage 19C090431_I012_MPCR
V-J pairing usage 19C090430_I012_MPCR
Frequency

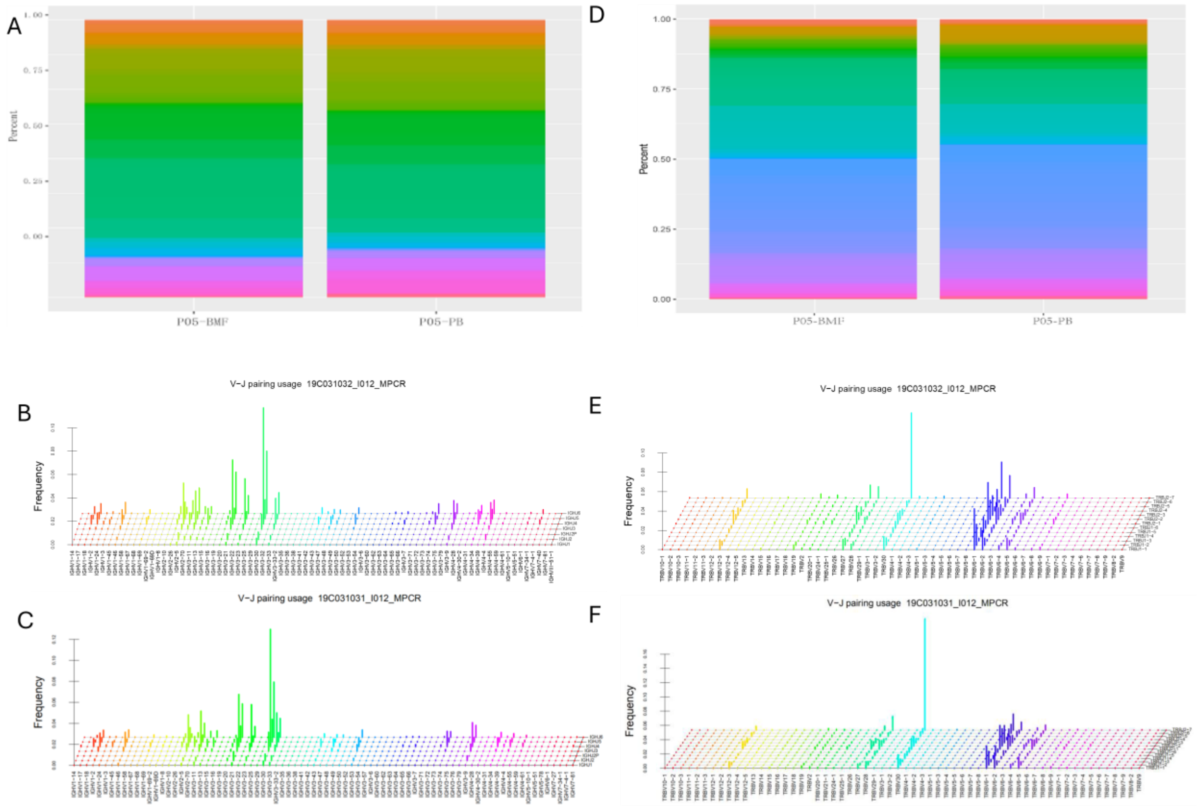

A
Percent
1.00
0.75
0.50
0.25
0.00
P05-BMF
P05-PB
B
V−J pairing usage 19C031032_I012_MPCR
Frequency
C
V−J pairing usage 19C031031_I012_MPCR
Frequency
D
Percent
1.00
0.75
0.50
0.25
0.00
P05-BMF
P05-PB
E
V−J pairing usage 19C031032_I012_MPCR
Frequency
F
V−J pairing usage 19C031031_I012_MPCR
Frequency

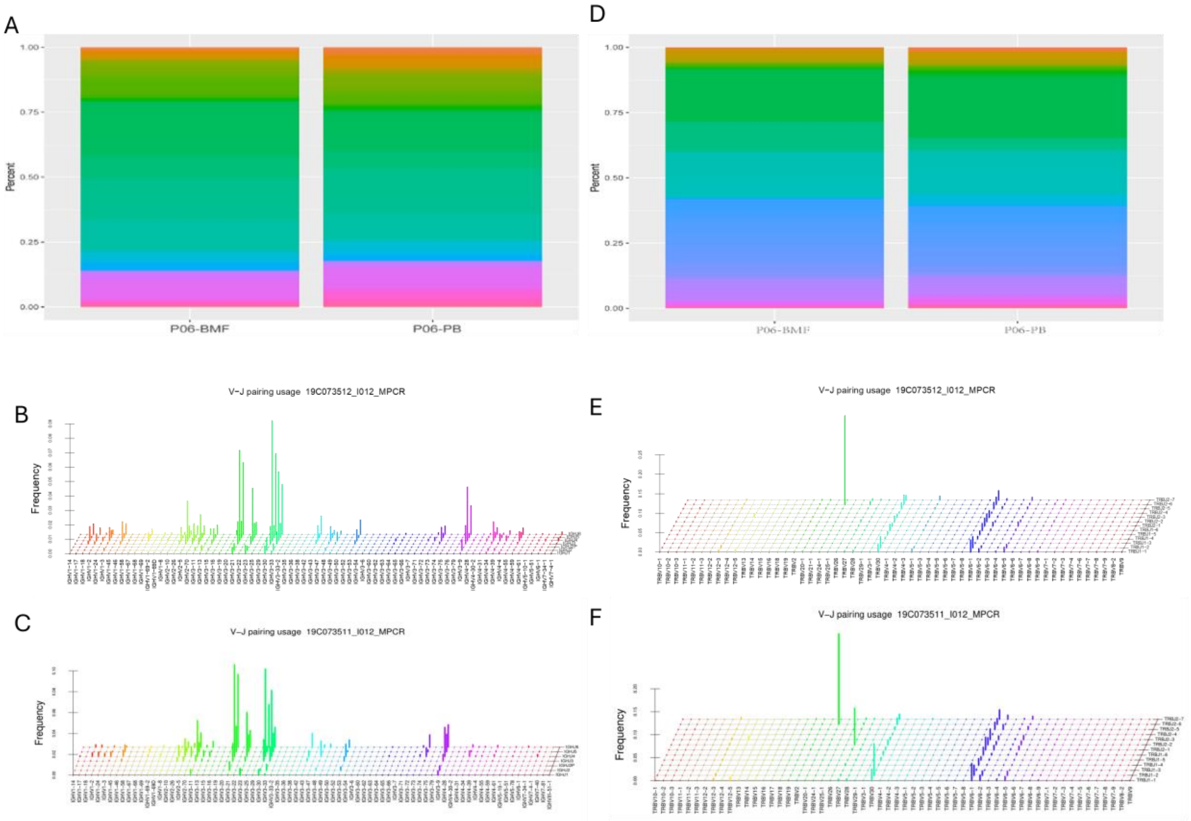

A
D
Percent
1.00
0.75
0.50
0.25
0.00
P06-BMF
P06-PB
B
E
V-J pairing usage 19C073512_I012_MPCR
Frequency
C
F
V-J pairing usage 19C073511_I012_MPCR

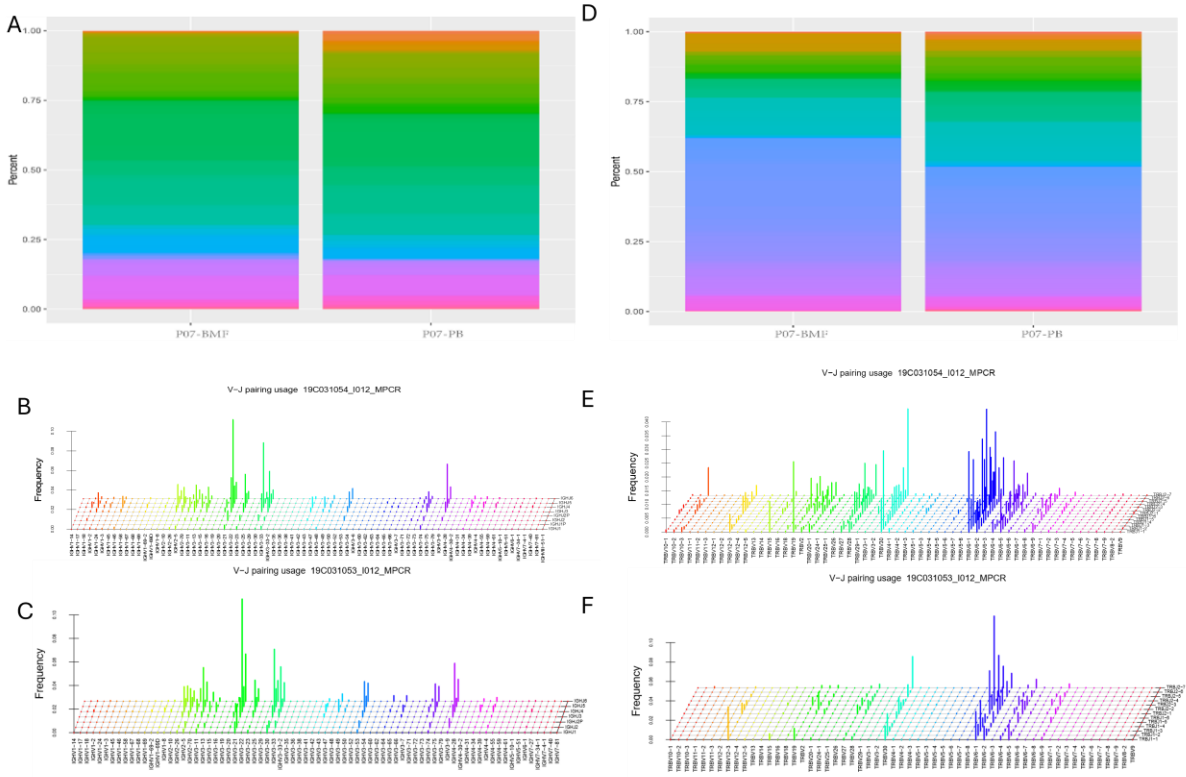
A
D
1.00
0.75
0.50
0.25
0.00
Percent
P07-BMF
P07-PB
B
C
E
F
V−J pairing usage 19C031054_I012_MPCR
V−J pairing usage 19C031053_I012_MPCR
Frequency

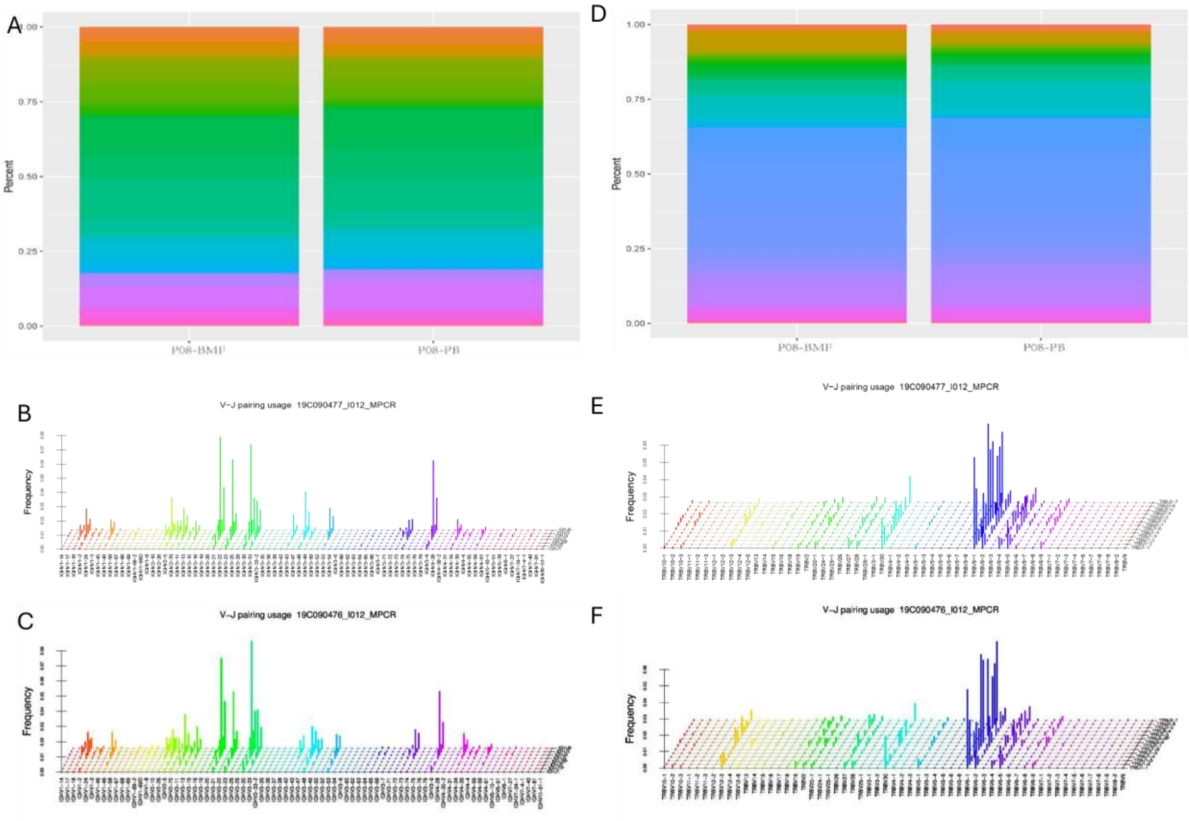
A
B
C
D
E
F
Percent
1.00
0.75
0.50
0.25
0.00
P08-BMF
P08-PB
V-J pairing usage 19C090477_I012_MPCR
V-J pairing usage 19C090476_I012_MPCR
Frequency

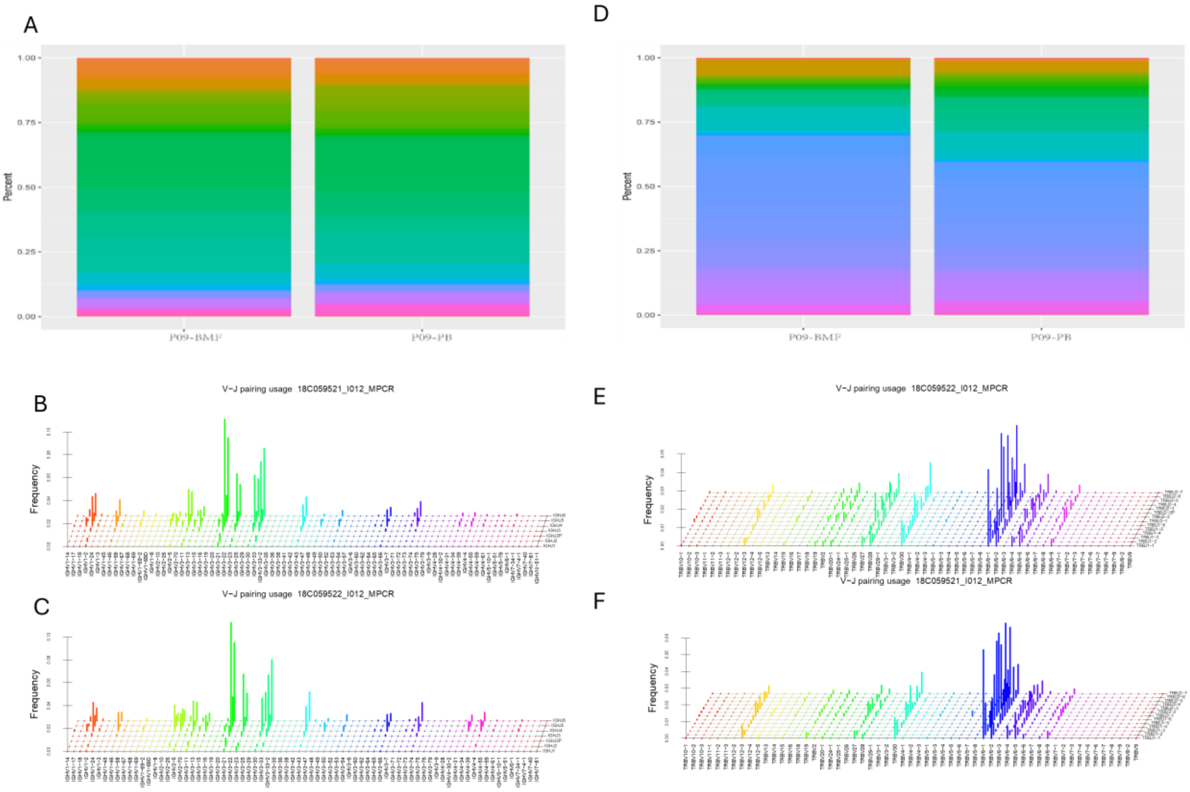
A
1.00
0.75
0.50
0.25
0.00
Percent
P09-BMF
P09-PB
D
1.00
0.75
0.50
0.25
0.00
Percent
P09-BMF
P09-PB
B
V-J pairing usage 18C059521_I012_MPCR
Frequency
C
V-J pairing usage 18C059522_I012_MPCR
Frequency
E
V-J pairing usage 18C059522_I012_MPCR
Frequency
F
V-J pairing usage 18C059521_I012_MPCR
Frequency

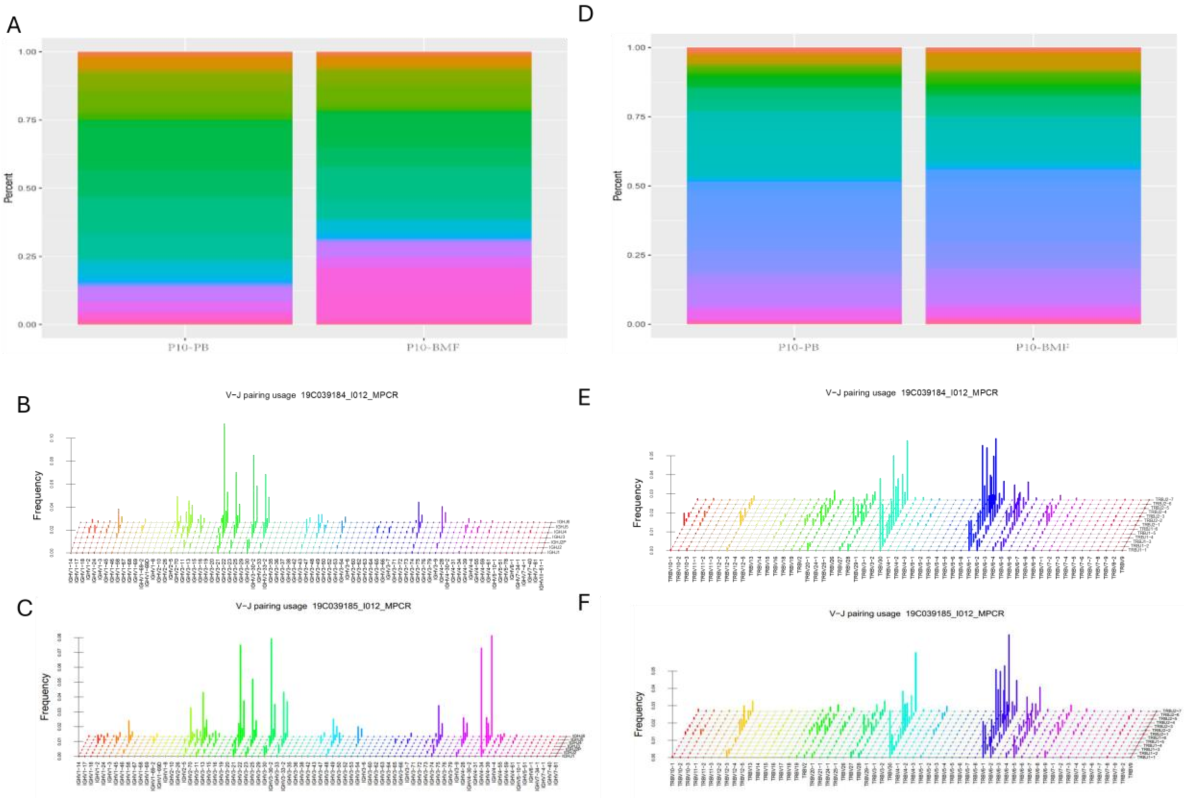

A
Percent
1.00
0.75
0.50
0.25
0.00
P10-PB
P10-BMF
D
Percent
1.00
0.75
0.50
0.25
0.00
P10-PB
P10-BMF
B
V−J pairing usage 19C039184_I012_MPCR
Frequency
E
V−J pairing usage 19C039184_I012_MPCR
Frequency
C
V−J pairing usage 19C039185_I012_MPCR
Frequency
F
V−J pairing usage 19C039185_I012_MPCR
Frequency

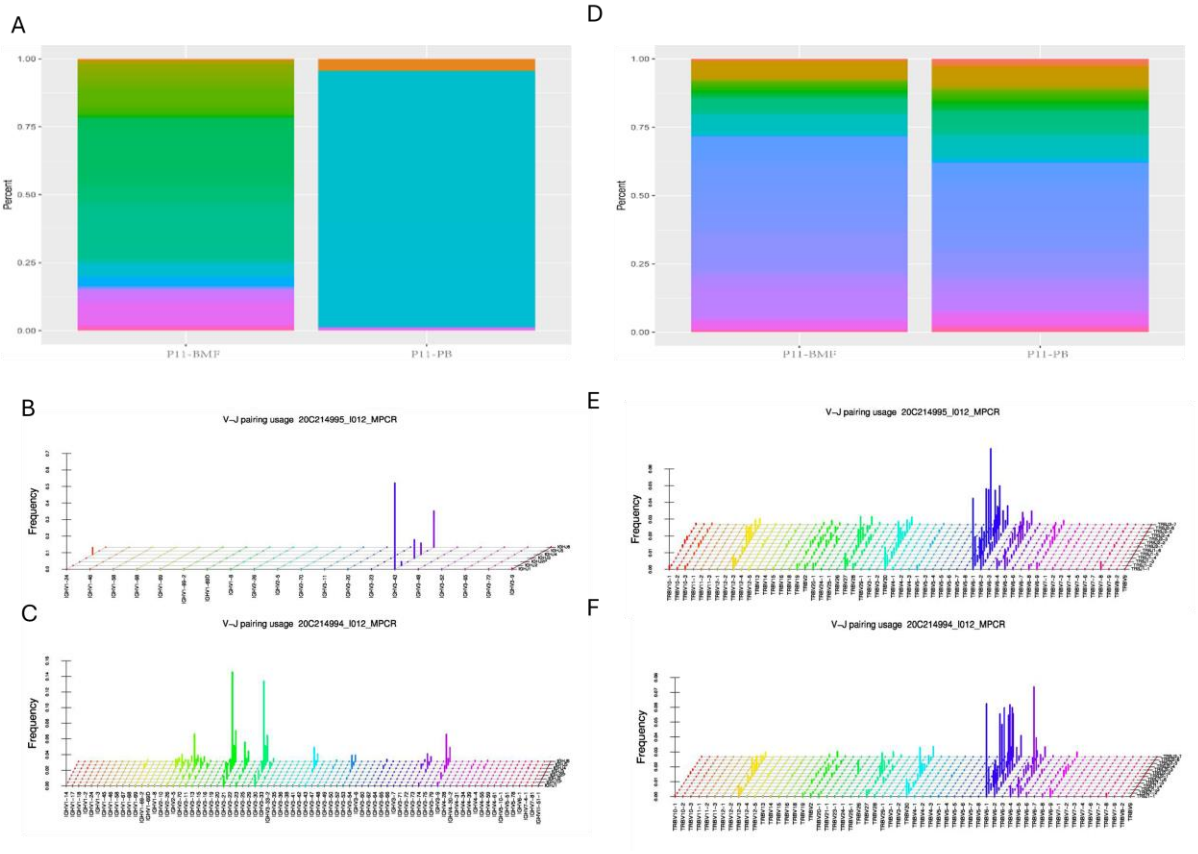
A
1.00
0.75
0.50
0.25
0.00
Percent
P11-BMF
P11-PB
D
1.00
0.75
0.50
0.25
0.00
Percent
P11-BMF
P11-PB
B
V-J pairing usage 20C214995_I012_MPCR
Frequency
C
V-J pairing usage 20C214994_I012_MPCR
Frequency
E
V-J pairing usage 20C214995_I012_MPCR
Frequency
F
V-J pairing usage 20C214994_I012_MPCR
Frequency

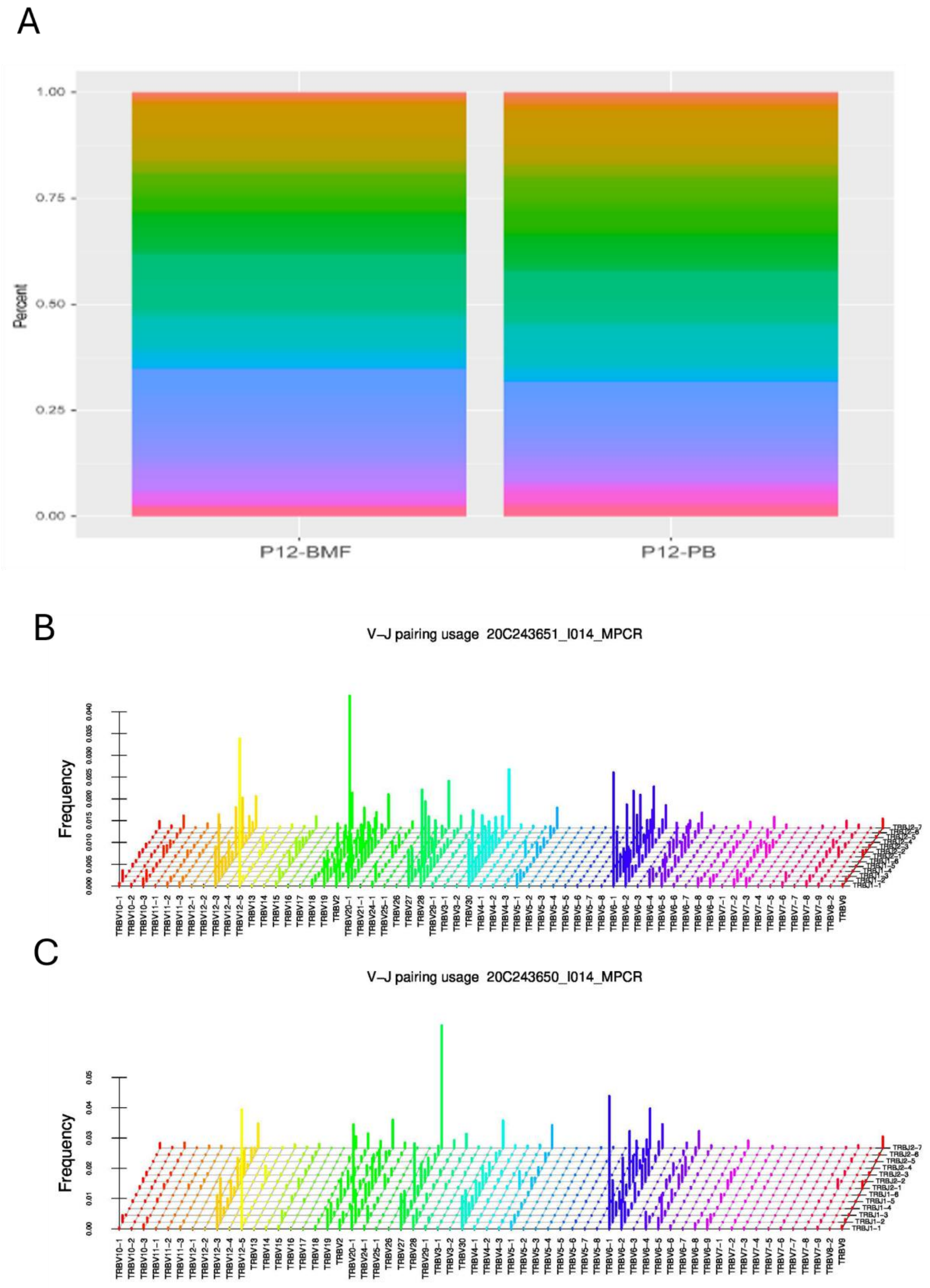
A
Percent
1.00
0.75
0.50
0.25
0.00
P12-BMF
P12-PB
B
V–J pairing usage 20C243651_I014_MPCR
Frequency
C
V–J pairing usage 20C243650_I014_MPCR
Frequency

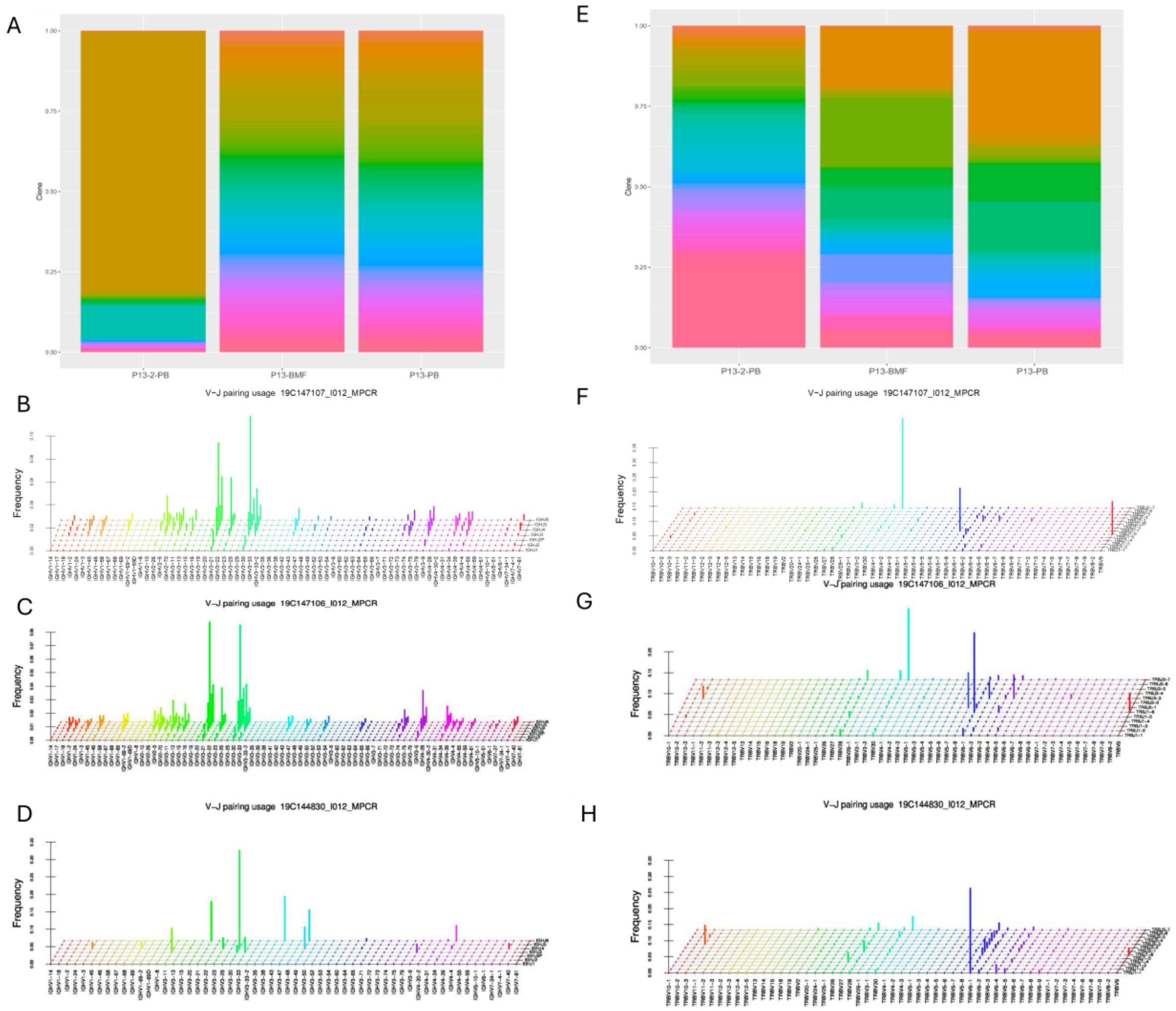
A
B
C
D
E
F
G
H
Clone
1.00
0.75
0.50
0.25
0.00
P13-2-PB
P13-BMF
P13-PB
V–J pairing usage 19C147107_I012_MPCR
V–J pairing usage 19C147106_I012_MPCR
V–J pairing usage 19C144830_I012_MPCR
Frequency

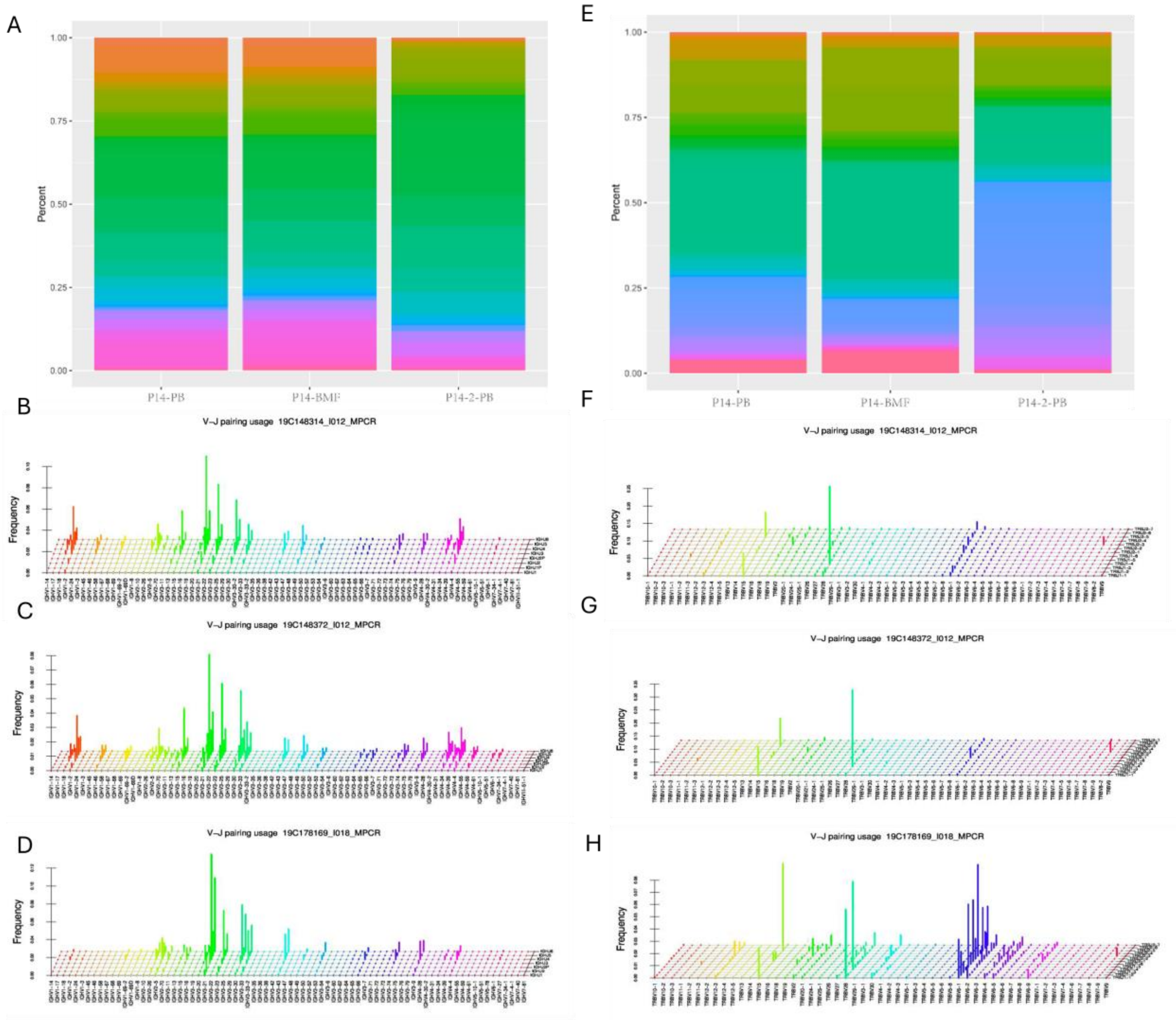
A
E
1.00
0.75
0.50
0.25
0.00
Percent
P14-PB
P14-BMF
P14-2-PB
B
V–J pairing usage 19C148314_I012_MPCR
Frequency
C
V–J pairing usage 19C148372_I012_MPCR
Frequency
D
V–J pairing usage 19C178169_I018_MPCR
Frequency
F
V–J pairing usage 19C148314_I012_MPCR
Frequency
G
V–J pairing usage 19C148372_I012_MPCR
Frequency
H
V–J pairing usage 19C178169_I018_MPCR
Frequency

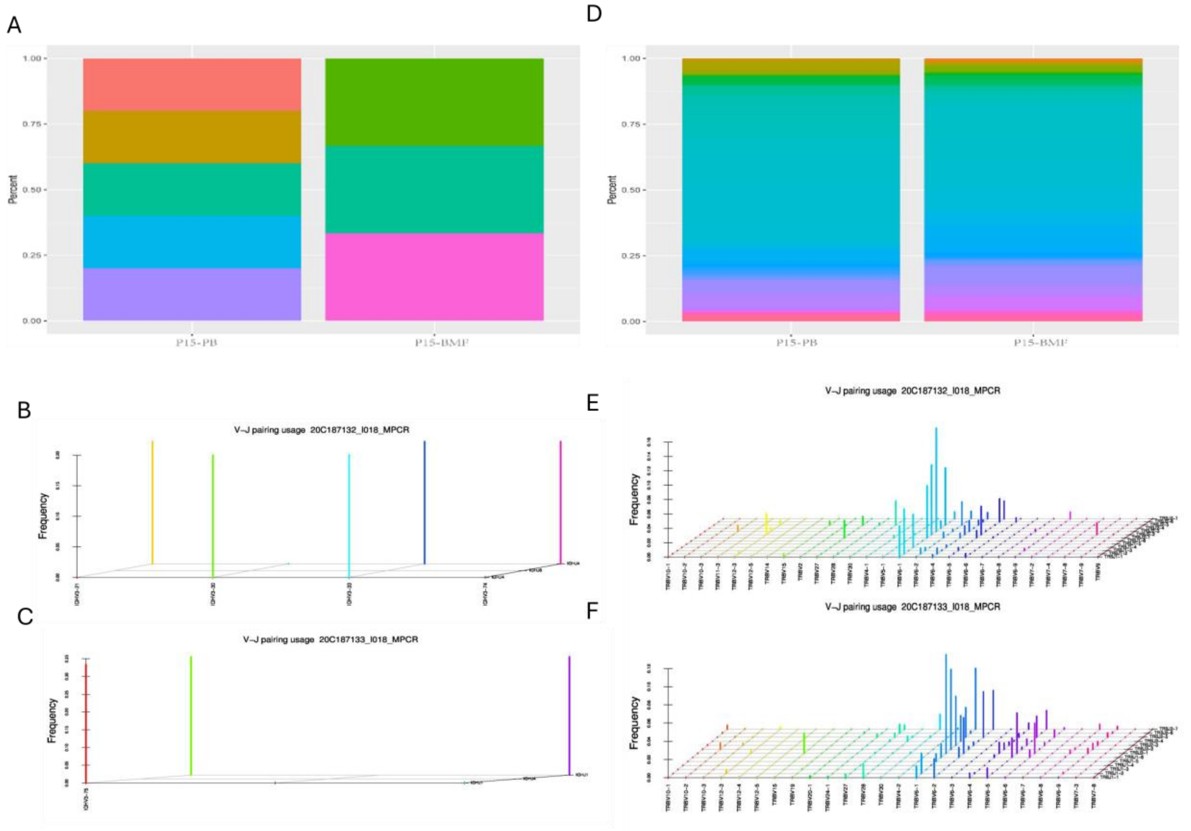
A
Percent
1.00
0.75
0.50
0.25
0.00
P15-PB
P15-BMF
B
V–J pairing usage 20C187132_I018_MPCR
Frequency
C
V–J pairing usage 20C187133_I018_MPCR
Frequency
D
Percent
1.00
0.75
0.50
0.25
0.00
P15-PB
P15-BMF
E
V–J pairing usage 20C187132_I018_MPCR
Frequency
F
V–J pairing usage 20C187133_I018_MPCR
Frequency

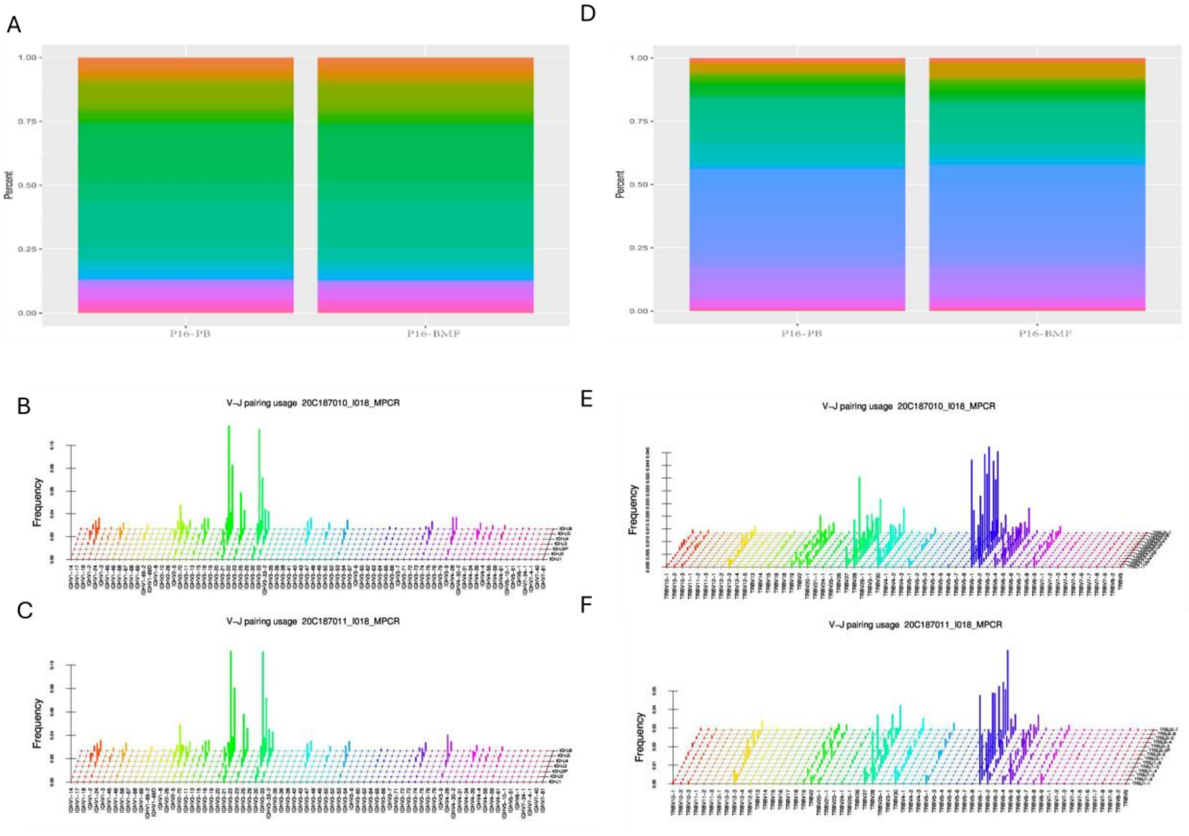
A
Percent
1.00
0.75
0.50
0.25
0.00
P16-PB
P16-BMF
D
Percent
1.00
0.75
0.50
0.25
0.00
P16-PB
P16-BMF
B
V-J pairing usage 20C187010_I018_MPCR
Frequency
E
V-J pairing usage 20C187010_I018_MPCR
Frequency
C
V-J pairing usage 20C187011_I018_MPCR
Frequency
F
V-J pairing usage 20C187011_I018_MPCR
Frequency

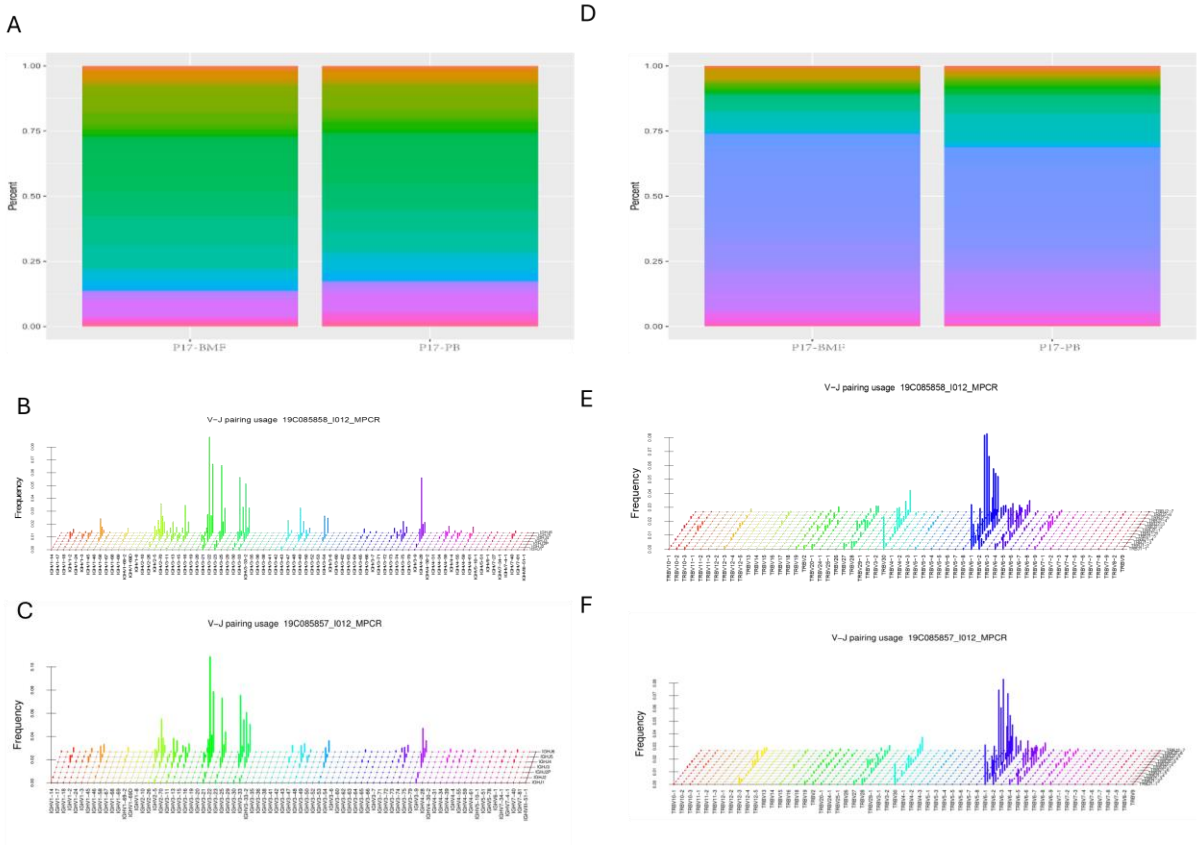
A
D
Percent
1.00
0.75
0.50
0.25
0.00
P17-BMF
P17-PB
B
E
C
F
V-J pairing usage 19C085858_I012_MPCR
V-J pairing usage 19C085857_I012_MPCR
Frequency
